# The Non-Catalytic Domains of *Drosophila* Katanin Regulate Its Abundance and Microtubule-Disassembly Activity

**DOI:** 10.1371/journal.pone.0123912

**Published:** 2015-04-17

**Authors:** Kyle D. Grode, Stephen L. Rogers

**Affiliations:** 1 Department of Biology, University of North Carolina at Chapel Hill, Chapel Hill, North Carolina, United States of America; 2 Carolina Center for Genome Sciences, University of North Carolina at Chapel Hill, Chapel Hill, North Carolina, United States of America; 3 Lineberger Comprehensive Cancer Center, University of North Carolina at Chapel Hill, Chapel Hill, North Carolina, United States of America; Institut de Génétique et Développement de Rennes, FRANCE

## Abstract

Microtubule severing is a biochemical reaction that generates an internal break in a microtubule and regulation of microtubule severing is critical for cellular processes such as ciliogenesis, morphogenesis, and meiosis and mitosis. Katanin is a conserved heterodimeric ATPase that severs and disassembles microtubules, but the molecular determinants for regulation of microtubule severing by katanin remain poorly defined. Here we show that the non-catalytic domains of *Drosophila* katanin regulate its abundance and activity in living cells. Our data indicate that the microtubule-interacting and trafficking (MIT) domain and adjacent linker region of the *Drosophila* katanin catalytic subunit Kat60 cooperate to regulate microtubule severing in two distinct ways. First, the MIT domain and linker region of Kat60 decrease its abundance by enhancing its proteasome-dependent degradation. The *Drosophila* katanin regulatory subunit Kat80, which is required to stabilize Kat60 in cells, conversely reduces the proteasome-dependent degradation of Kat60. Second, the MIT domain and linker region of Kat60 augment its microtubule-disassembly activity by enhancing its association with microtubules. On the basis of our data, we propose that the non-catalytic domains of *Drosophila* katanin serve as the principal sites of integration of regulatory inputs, thereby controlling its ability to sever and disassemble microtubules.

## Introduction

Microtubules are cytoskeletal polymers composed of tubulin heterodimers that form complex and highly organized arrays with diverse functions during the cell cycle [1]. Microtubules during interphase, for example, form the axonemal structures of cilia and flagella, the bundled arrays of neuronal cells, and the web-like networks of epithelial cells. Microtubule superstructures such as these are required, respectively, for ciliary and flagellar motility, directional molecular transport, and the establishment and maintenance of cytoplasmic polarity [2–4]. Microtubules during cell division, by contrast, create a single higher-order structure called a bipolar spindle that is required for the segregation of genetic material within the germline and somatic tissues of multi-cellular organisms [5,6]. Despite their organizational and functional diversity during the cell cycle, microtubule superstructures are built and remodeled by many of the same microtubule-stabilizing and destabilizing activities. One activity that is important for the proper organization and function of microtubule superstructures throughout the cell cycle is the severing of microtubules [7,8]. Microtubule severing is a biochemical reaction that generates an internal break in a microtubule and catalysis of microtubule severing is mediated by a small family of ATPases called microtubule-severing enzymes. To date, three microtubule-severing enzymes have been identified—katanin, spastin, and fidgetin—and each contains a single catalytic AAA ATPase domain that is highly conserved between these enzymes [7]. Recent studies have uncovered diverse cellular roles for the microtubule-severing enzyme family and these roles are highlighted by the wide range of disorders and diseases that are associated with their mutations such as infertility, microphthalmia, and hereditary spastic paraplegia [9–12]. Central to the cellular roles of the microtubule-severing enzymes is the control of their abundance and activity, however the regulatory mechanisms that coordinate microtubule severing with the normal cellular program remain poorly understood.

Katanin was first purified from sea urchin eggs based on its ATP-dependent microtubule-severing activity and it was the first protein identified that severed and disassembled microtubules [13]. Katanin is a heterodimer consisting of a catalytic subunit and a regulatory subunit and conserved homologues of both katanin subunits are widely represented in eukaryotes. All canonical katanin catalytic subunits exhibit a bipartite domain structure with an NH_2_-terminal microtubule-interacting and trafficking (MIT) domain connected to a COOH-terminal AAA ATPase domain via a poorly conserved linker region [7]. The nuclear magnetic resonance structure of the MIT domain from the mouse katanin catalytic subunit KATNA1 revealed that it consists of a three-helix bundle with many potential protein-protein interaction surfaces [14]. Biochemical studies of katanin catalytic subunits from several species have shown that the MIT domain is necessary for binding the katanin regulatory subunit [15,16] and that the MIT domain together with the linker region is sufficient for binding both the katanin regulatory subunit [16] and microtubules [16–19]. Based on recent x-ray crystallographic studies of spastin [20,21], the AAA ATPase domain of katanin catalytic subunits likely comprises a canonical α/β nucleotide-binding domain surrounded by structural elements involved in tubulin-binding and oligomerization. Mutagenesis studies of katanin catalytic subunits from several species have established that ATP hydrolysis by the AAA domain is absolutely required for severing microtubules [15,22–24]. Similar to katanin catalytic subunits, all canonical katanin regulatory subunits exhibit a bipartite domain structure, but with an NH_2_-terminal WD40 domain connected to a conserved COOH-terminal domain (CTD) via a proline-rich linker region [7]. The WD40 domain of katanin regulatory subunits typically consists of six WD40 repeats that are predicted to fold into a six-bladed β-propeller architecture, whereas the CTD of katanin regulatory subunits is predicted to be mostly α-helical. Biochemical studies of katanin regulatory subunits from several species have demonstrated that the CTD is necessary for binding the katanin catalytic subunit [25] and sufficient for binding both the katanin catalytic subunit [15,25,26] and microtubules [15]. Thus, katanin is thought to form a ring-shaped complex with non-catalytic microtubule-binding sites distributed between its subunits that extend radially from its central ATP-fueled motor. This quaternary structure suggests that the non-catalytic domains of katanin are crucial to its microtubule-severing activity, however this hypothesis has not been rigorously tested by any functional study to date.

Based on their observation that the katanin catalytic subunit forms transient hexamers only in the presence of ATP and microtubules, Hartman and Vale proposed a model—hereafter referred to as the katanin assembly model—for how katanin severs and disassembles microtubules [17]. In this model, katanin heterodimers are monomeric in the ADP-bound state, but nucleotide exchange for ATP and microtubule-binding increases inter-subunit binding affinity, leading to oligomerization. Once assembled, the katanin complex binds the microtubule polymer with high affinity due to the formation of multiple contacts with tubulin and its ATPase activity is stimulated, resulting in nucleotide-driven conformational changes that sever the microtubule polymer. Thus, the katanin assembly model postulates that the oligomerization of katanin into a catalytically active complex occurs in a concentration- and microtubule-dependent manner via multivalent interaction with the microtubule polymer. The prediction of this model for the roles of the non-catalytic domains of katanin is that they enhance the initial targeting of its subunits to the microtubule polymer and/or influence the subsequent balancing between subunit-microtubule and inter-subunit interactions. Although recent biophysical studies have advanced our understanding of the katanin assembly model [19,24,27,28], the relative contribution of the non-catalytic domains of katanin to its microtubule-severing activity in vivo remains unknown. Here we conduct a structure-function analysis of *Drosophila* katanin in cultured *Drosophila* S2 cells to test the hypothesis that the non-catalytic domains of katanin make distinct contributions to microtubule severing in living cells.

## Results and Discussion

### Cultured *Drosophila* S2 cells overexpressing Kat60 exhibit microtubule-disassembly in a concentration-dependent manner

**Fig 1:**
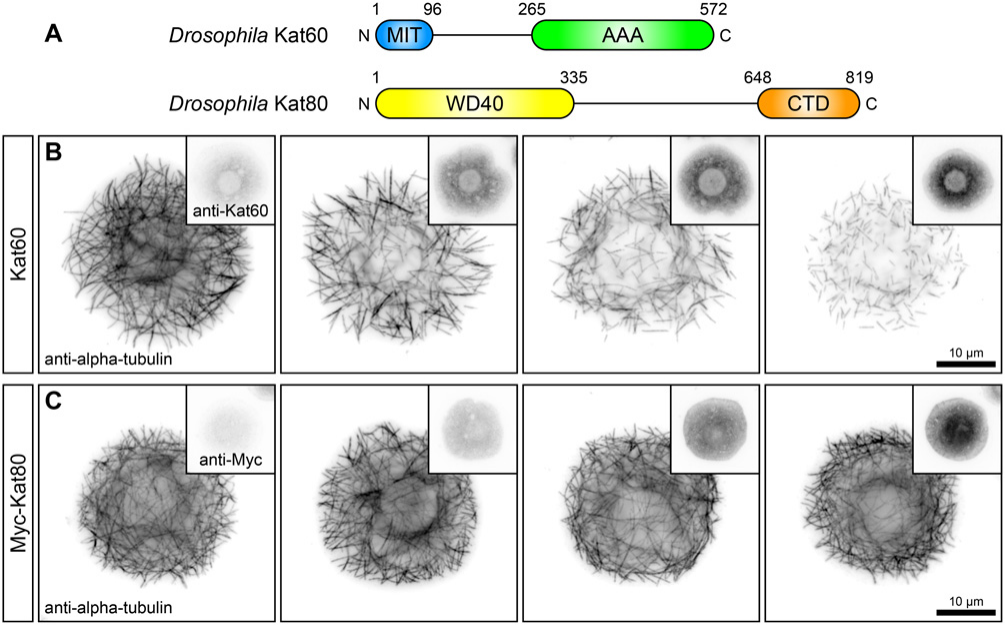
Cultured *Drosophila* S2 cells overexpressing Kat60 exhibit microtubule-disassembly in a concentration-dependent manner. (A) Schematic of the domain structure of the *Drosophila* canonical katanin catalytic subunit Kat60 (Top) and the canonical katanin regulatory subunit Kat80 (Bottom). (B and C) Immunofluorescence microscopy images of *Drosophila* S2 cells overexpressing Kat60 (B) or Myc-Kat80 (C) and immunostained for alpha-tubulin and Kat60 (B) or Myc (C). (B) A cell not overexpressing Kat60 (First) and cells overexpressing Kat60 (Second-Fourth). (C) A cell not overexpressing Myc-Kat80 (First) and cells overexpressing Myc-Kat80 (Second-Fourth). Alpha-tubulin, Kat60, and Myc images in each panel are displayed with the same scaling.

The *Drosophila* genome contains single genes that encode the canonical katanin catalytic subunit, Kat60, and the canonical katanin regulatory subunit, Kat80 (Fig 1A). Previous studies have shown that Kat60 by itself can sever and disassemble microtubules in vitro [23,24] and that overexpression of Kat60 is sufficient to promote microtubule disassembly in cultured S2 cells [29] and in larval muscle cells [30]. To date, there have been no functional studies of Kat80. To begin our structure-function analysis of the microtubule-severing activity of *Drosophila* katanin, we first overexpressed Kat60 in S2 cells and examined the effects on microtubules by immunofluorescence microscopy. Cells overexpressing Kat60 displayed a disorganized array of fragmented microtubules and the overall length of microtubules appeared to decrease with increasing levels of Kat60 (Fig 1B). Many of these cells contained individual microtubules with both ends visible and several of these cells had only short microtubule fragments; cells that showed a complete loss of microtubules were never observed. In addition, cells overexpressing Kat60 had reduced levels of alpha-tubulin compared to control cells and the degree of reduction increased with increasing levels of Kat60. The inverse relationship between alpha-tubulin and Kat60 levels indicates that cells overexpressing Kat60 exhibit microtubule disassembly in a concentration-dependent manner and the disorganized array of short microtubules suggests that this disassembly is due to the microtubule-severing activity of Kat60. The effects of overexpression of Kat60 on microtubules and alpha-tubulin levels in S2 cells are similar to those observed in other animal cell types overexpressing the katanin catalytic subunit [15,31]. To determine if Kat80 possesses microtubule-disassembly activity, we overexpressed Myc-tagged Kat80 (Myc-Kat80) in cells and examined the effects on microtubules by immunofluorescence microscopy. Cells overexpressing Myc-Kat80 did not exhibit microtubule fragmentation or have reduced levels of alpha-tubulin (Fig 1C), consistent with a previous report that the sea urchin katanin regulatory subunit KATNB1 cannot sever and disassemble microtubules in vitro [25]. Taken together, these findings demonstrate that overexpression of Kat60, but not Kat80, is sufficient to promote microtubule disassembly in S2 cells and that the loss of microtubules in these cells occurs as a function of Kat60 levels.

### Development of a single-cell assay to measure the microtubule-disassembly activity of Kat60 using automated microscopy

To quantitate the effects of overexpression of Kat60 on microtubules in cells, we developed a high-content immunofluorescence microscopy assay to measure the loss of microtubules as a function of Kat60 levels. For this assay, we generated cells that stably express 1) GFP under the control of a constitutive promoter and 2) versions of Kat60 and Kat80, either alone or in combination, under the control of a copper-inducible promoter. In this assay, we first induce cells with CuSO_4_ and then we acquire images of tens of thousands of cells stained for DNA and immunostained for alpha-tubulin and Kat60 using automated microscopy (S1 Fig). Next, we process and analyze the images using the open-source CellProfiler software [32]. The DNA images allow us to first identify nuclei, which then permits the use of the GFP images to identify individual cells and create cell outlines. Finally, we extract the average alpha-tubulin and Kat60 immunofluorescence intensities from each outlined cell based on the alpha-tubulin and Kat60 images, respectively.

**Fig 2:**
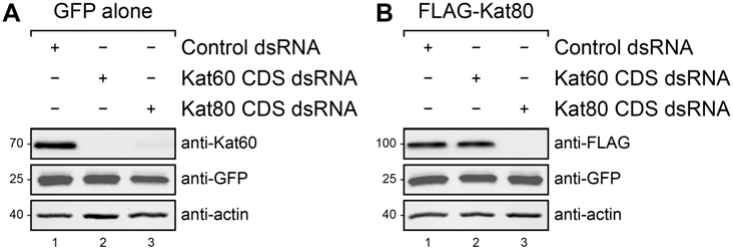
Depletion of Kat80 reduces steady-state Kat60 levels in cells. (A and B) Immunoblots of *Drosophila* S2 cell lysates prepared from cells stably expressing GFP alone (A) or GFP and copper-inducible FLAG-Kat80 (B) that were treated with control (lane 1), Kat60 CDS (lane 2), or Kat80 CDS dsRNA (lane 3) for 7 days total. The cells described in B were also treated with 0.1 mM CuSO_4_ for 20 hours. Molecular weights (in Kd) are shown on the left.

To minimize the potential contribution of endogenous Kat60 and Kat80 to the single-cell measurements collected in each experiment, we devised an RNA interference (RNAi) strategy to specifically deplete the endogenous proteins from cells using double-stranded RNAs (dsRNA) that target the untranslated regions (UTR) of Kat60 and Kat80. Before evaluating the efficacy of this strategy, we first sought to determine if dsRNAs targeting the coding sequences (CDS) of Kat60 and Kat80 are effective in eliminating both proteins from cells. As an initial step, we treated cells stably expressing GFP alone with Kat60 or Kat80 CDS dsRNA and analyzed the steady-state levels of Kat60 by immunoblotting. Endogenous Kat60 was undetectable in cells treated with Kat60 CDS dsRNA and the levels of Kat60 in cells treated with Kat80 CDS dsRNA were dramatically reduced compared to those in cells treated with control dsRNA (Fig 2A), suggesting that Kat80 functions to stabilize Kat60. To verify the effectiveness of Kat80 CDS dsRNA treatment, we treated cells stably expressing GFP and copper-inducible FLAG-tagged Kat80 (FLAG-Kat80) with Kat60 or Kat80 CDS dsRNA prior to induction and analyzed the levels of FLAG-Kat80 by immunoblotting. FLAG-Kat80 was undetectable in cells treated with Kat80 CDS dsRNA and the levels of FLAG-Kat80 in cells treated with Kat60 CDS dsRNA were indistinguishable from those in cells treated with control dsRNA (Fig 2B). Thus, in addition to demonstrating that Kat60 and Kat80 CDS dsRNA treatments are effective in eliminating both proteins from cells, these findings provide strong evidence that Kat80 is required to stabilize Kat60 in cells. To our knowledge, the loss of Kat60 upon depletion of Kat80 represents the first example of instability of one katanin subunit in the absence of the other subunit.

Having observed that both Kat60 and Kat80 CDS dsRNA treatments reduce steady-state Kat60 levels in cells, we next tested the effectiveness of Kat60 and Kat80 UTR dsRNA treatments in depleting the endogenous proteins from cells. To do this, we treated cells stably expressing GFP alone with Kat60 and Kat80 UTR dsRNA, either alone or in combination, and analyzed the steady-state levels of Kat60 by immunoblotting. The levels of Kat60 in cells treated with Kat60 and Kat80 UTR dsRNA, either alone or in combination, were dramatically reduced compared to those in cells treated with control dsRNA (S2 Fig). From these results, we conclude that Kat60 and Kat80 UTR dsRNA treatments effectively deplete the endogenous proteins from cells and unless otherwise noted, we employed our Kat60 and Kat80 UTR RNAi strategy in all subsequent experiments in this study.

To investigate the effects of depletion of Kat60 and Kat80 on the single-cell measurements collected in each experiment, we used our assay to measure the steady-state levels of alpha-tubulin and Kat60 in cells stably expressing GFP alone that were treated with both Kat60 and Kat80 UTR dsRNA. These cells had only slightly reduced levels of alpha-tubulin compared to cells treated with control dsRNA (S3 Fig), indicating that depletion of Kat60 and Kat80 causes minimal perturbation of steady-state alpha-tubulin levels in cells. As expected, these cells had reduced levels of Kat60 compared to cells treated with control dsRNA, demonstrating that a difference in Kat60 levels between control and Kat60 and Kat80 depleted cells can be measured using our assay. Based on such an approximation of endogenous Kat60 levels, we can estimate the fold overexpression levels of Kat60 in cells expressing Kat60 to provide a biological context for the single-cell measurements collected in each experiment.

**Fig 3:**
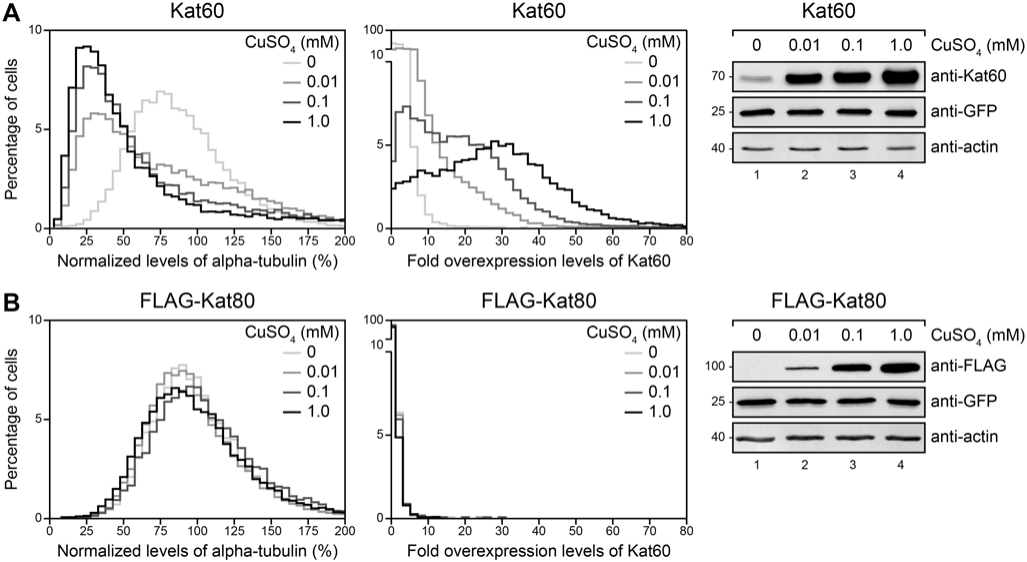
Inducible expression of Kat60 results in measurable microtubule disassembly in our single-cell assay. (A and B) Histograms of normalized levels of alpha-tubulin (Left) and fold overexpression levels of Kat60 (Middle) in *Drosophila* S2 cells stably expressing GFP and copper-inducible Kat60 (A) or FLAG-Kat80 (B) that were treated with both Kat60 and Kat80 UTR dsRNA for 7 days total. The cells described in A and B were also treated with 0 (light gray), 0.01 (medium gray), 0.1 (dark gray), or 1.0 mM CuSO_4_ (black) for 20 hours and immunostained for alpha-tubulin and Kat60. Normalized levels of alpha-tubulin are expressed as a percentage of the mean levels of alpha-tubulin in cells stably expressing GFP alone that were treated with both Kat60 and Kat80 UTR dsRNA for 7 days total. Fold overexpression levels of Kat60 are expressed as a fraction of the difference in the mean levels of Kat60 between cells stably expressing GFP alone that were treated with control and both Kat60 and Kat80 UTR dsRNA for 7 days total. Data are pooled from three independent experiments (see S1 Table for summary statistics of the single-cell measurements collected). (Right) Immunoblots of cell lysates prepared from the cells described in A and B. Molecular weights (in Kd) are shown on the left.

To establish baseline single-cell measurements for the effects of inducible expression of Kat60 and Kat80 on microtubules, we used our assay to measure the loss of microtubules in cells stably expressing GFP and copper-inducible Kat60 or FLAG-Kat80 that were induced with increasing concentrations of CuSO_4_. Cells induced to express Kat60 had reduced levels of alpha-tubulin compared to control cells and the degree of reduction increased with increasing concentrations of CuSO_4_ over a 100-fold CuSO_4_ concentration range (Fig 3A and S1 Table). Appropriately, Kat60 accumulated at levels that increased with increasing concentrations of CuSO_4_ in these cells. As expected, cells induced to express FLAG-Kat80 had almost identical levels of alpha-tubulin and Kat60 relative to control cells and FLAG-Kat80 accumulated at levels that increased with increasing concentrations of CuSO_4_ in these cells (Fig 3B and S1 Table). Collectively, these data demonstrate that inducible expression of Kat60, but not Kat80, results in measurable microtubule disassembly in our single-cell assay.

### The MIT domain of Kat60 is dispensable for its microtubule-disassembly activity at high levels of accumulation in cells

**Fig 4:**
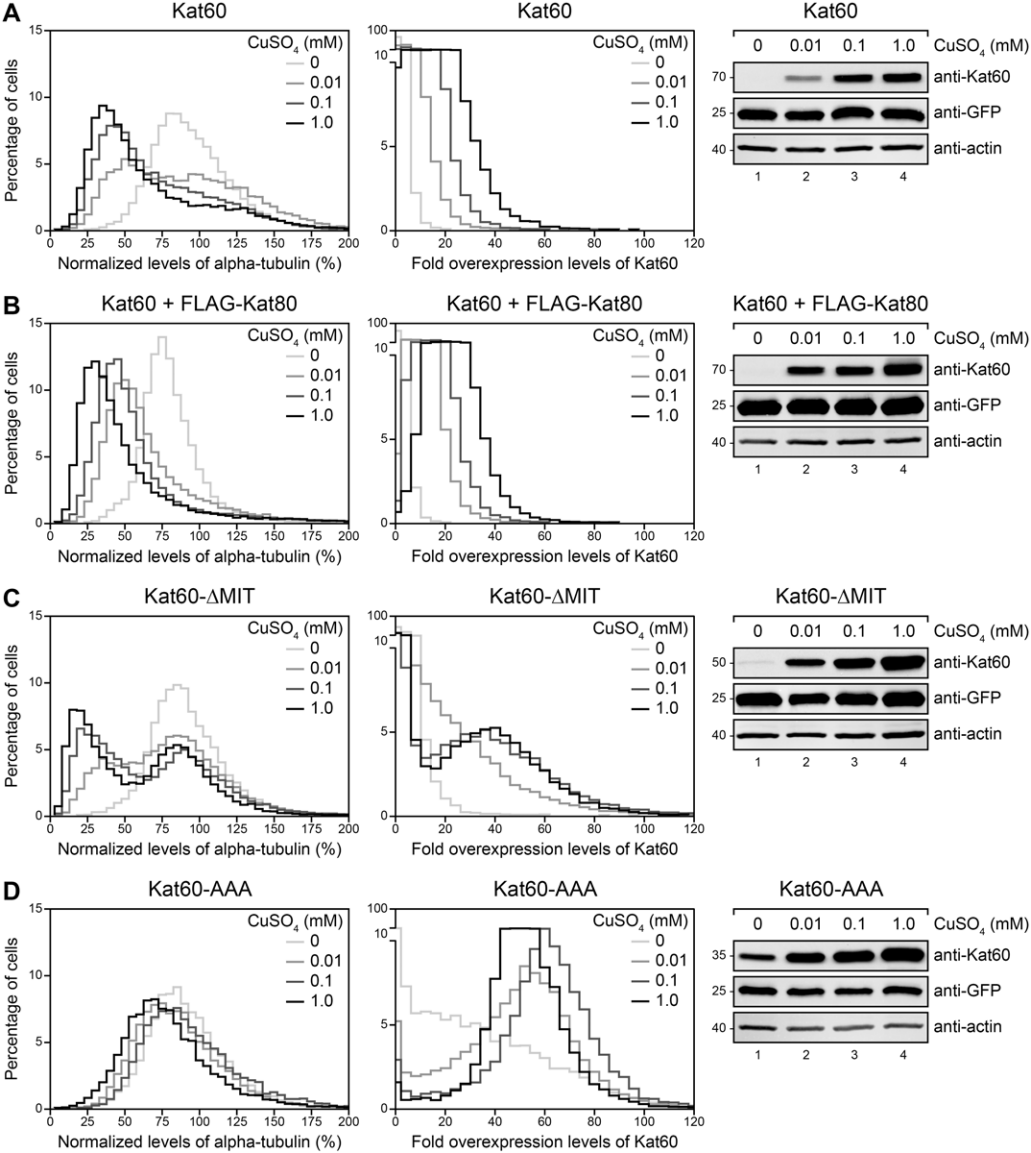
Kat60 lacking the MIT domain disassembles microtubules at high levels of accumulation in cells. (A-D) Histograms of normalized levels of alpha-tubulin (Left) and fold overexpression levels of Kat60 (Middle) in *Drosophila* S2 cells stably expressing GFP and copper-inducible Kat60 (A), Kat60 and FLAG-Kat80 (B), Kat60-ΔMIT (C), or Kat60-AAA (D) that were treated with both Kat60 and Kat80 UTR dsRNA for 7 days total. The cells described in A-D were also treated with 0 (light gray), 0.01 (medium gray), 0.1 (dark gray), or 1.0 mM CuSO_4_ (black) for 20 hours and immunostained for alpha-tubulin and Kat60. Normalized levels of alpha-tubulin are expressed as a percentage of the mean levels of alpha-tubulin in cells stably expressing GFP alone that were treated with both Kat60 and Kat80 UTR dsRNA for 7 days total. Fold overexpression levels of Kat60 are expressed as a fraction of the difference in the mean levels of Kat60 between cells stably expressing GFP alone that were treated with control and both Kat60 and Kat80 UTR dsRNA for 7 days total. Data are pooled from three independent experiments (see S2 Table for summary statistics of the single-cell measurements collected). (Right) Immunoblots of cell lysates prepared from the cells described in A-D. Molecular weights (in Kd) are shown on the left.

To assess the contribution of the non-catalytic domains of *Drosophila* katanin to its microtubule-disassembly activity, we used our assay to measure the loss of microtubules in cells induced to express Kat60 alone or together with FLAG-Kat80, Kat60 lacking the MIT domain (Kat60-ΔMIT), or Kat60 lacking the MIT domain and linker region (Kat60-AAA). Compared to cells induced to express Kat60 alone, cells induced to express Kat60 together with FLAG-Kat80 had reduced levels of alpha-tubulin (Fig 4A and 4B and S2 Table), suggesting that Kat80 affects the microtubule-disassembly activity of Kat60. However, uninduced cells also had reduced levels of alpha-tubulin, thereby complicating an assessment of the contribution of Kat80 to the microtubule-disassembly activity of Kat60. In support of the notion that Kat80 increases the abundance of Kat60, Kat60 in the presence of FLAG-Kat80 accumulated at slightly higher levels than Kat60 alone in cells. Surprisingly, cells induced to express Kat60-ΔMIT either did not have reduced levels of alpha-tubulin or they had reduced levels of alpha-tubulin similar to cells induced to express Kat60 (Fig 4A and 4C and S2 Table), indicating that the MIT domain of Kat60 is, at the very least, dispensable for its microtubule-disassembly activity. Coincidentally, Kat60-ΔMIT either did not accumulate at detectable levels or it accumulated at notably higher levels than Kat60 in cells, suggesting that once expressed, the MIT domain of Kat60 functions to decrease its abundance. Thus, in addition to providing strong evidence that the MIT domain of Kat60 is dispensable for its microtubule-disassembly activity at high levels of accumulation, these results point to the need for alternative strategies to accurately assess the importance of the MIT domain of Kat60 to its activity. Consistent with the hypothesis that the non-catalytic domains of katanin are crucial to its microtubule-disassembly activity, cells induced to express Kat60-AAA did not have reduced levels of alpha-tubulin relative to cells induced to express Kat60 (Fig 4A and 4D and S2 Table). Remarkably, Kat60-AAA accumulated at higher levels than Kat60-ΔMIT in cells, strongly suggesting that the MIT domain and linker region of Kat60 cooperate to decrease its abundance.

### The MIT domain and linker region of Kat60 are required for its proteasome-dependent degradation in cells

**Fig 5:**
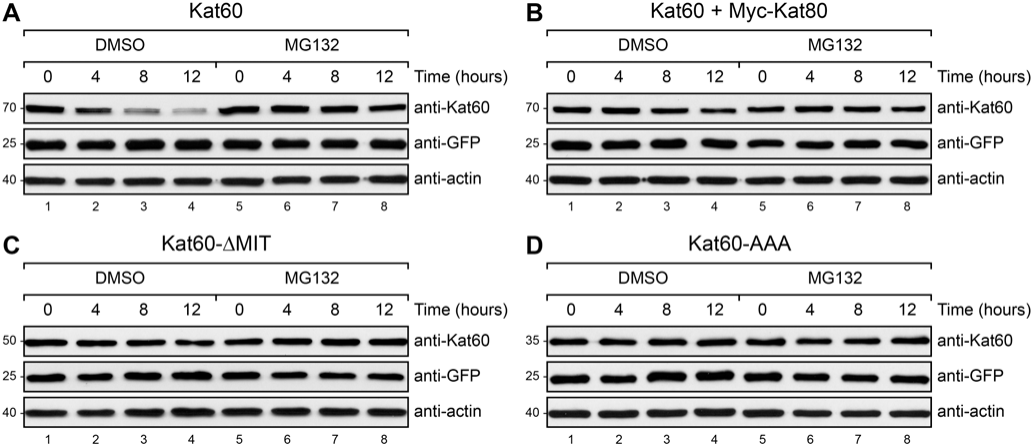
Kat60 lacking the MIT domain or the MIT domain and linker region is not detectably degraded in cells. (A-D) Immunoblots of *Drosophila* S2 cell lysates prepared from cells stably expressing GFP and copper-inducible Kat60 (A), Kat60 and Myc-Kat80 (B), Kat60-ΔMIT (C), or Kat60-AAA (D) that were treated with both Kat60 and Kat80 UTR dsRNA for 7 days total. The cells described in A-D were also treated with 1.0 (A), 1.0 (B), 0.1 (C), or 0.01 mM CuSO_4_ (D) for 16 hours, washed with S2M, and treated with DMSO (lanes 1–4) or 50 μM MG132 (lanes 5–8) for 0 (lanes 1 and 5), 4 (lanes 2 and 6), 8 (lanes 3 and 7), or 12 hours (lanes 4–8). Molecular weights (in Kd) are shown on the left.

Given our unexpected findings that both Kat60-ΔMIT and Kat60-AAA accumulate at higher levels than Kat60 in cells, we next explored how the non-catalytic domains of *Drosophila* katanin regulate its abundance. Because katanin catalytic subunits from several species have been shown to be ubiquitinated by ubiquitin ligase complexes [33–36] and degraded by the proteasome [36,37], we speculated that the non-catalytic domains of *Drosophila* katanin regulate its abundance by affecting its degradation via the ubiquitin-proteasome system. To test this hypothesis, we first pulsed the expression of Kat60 alone or together with Myc-Kat80, Kat60-ΔMIT, or Kat60-AAA in cells and then we used immunoblotting to analyze the degradation of these proteins in cells treated with DMSO or the 26S proteasome inhibitor MG132. Whereas Kat60 was markedly degraded in control cells, it was not detectably degraded in cells treated with MG132 (Fig 5A), demonstrating that the degradation of Kat60 is proteasome-dependent. In contrast to Kat60 alone, Kat60 in the presence of Myc-Kat80 was only slightly degraded in control cells (Fig 5B), indicating that Kat80 reduces the proteasome-dependent degradation of Kat60. Strikingly, both Kat60-ΔMIT and Kat60-AAA were not detectably degraded in control cells (Fig 5C and 5D), providing strong evidence that the MIT domain and linker region of Kat60 are required for its proteasome-dependent degradation. From these results, we conclude that the MIT domain and linker region of Kat60 decrease its abundance by enhancing its proteasome-dependent degradation and that Kat80 conversely increases the abundance of Kat60 by reducing its proteasome-dependent degradation.

### The MIT domain of Kat60 is important for its ability to disassemble microtubules at low levels of accumulation in cells

**Fig 6:**
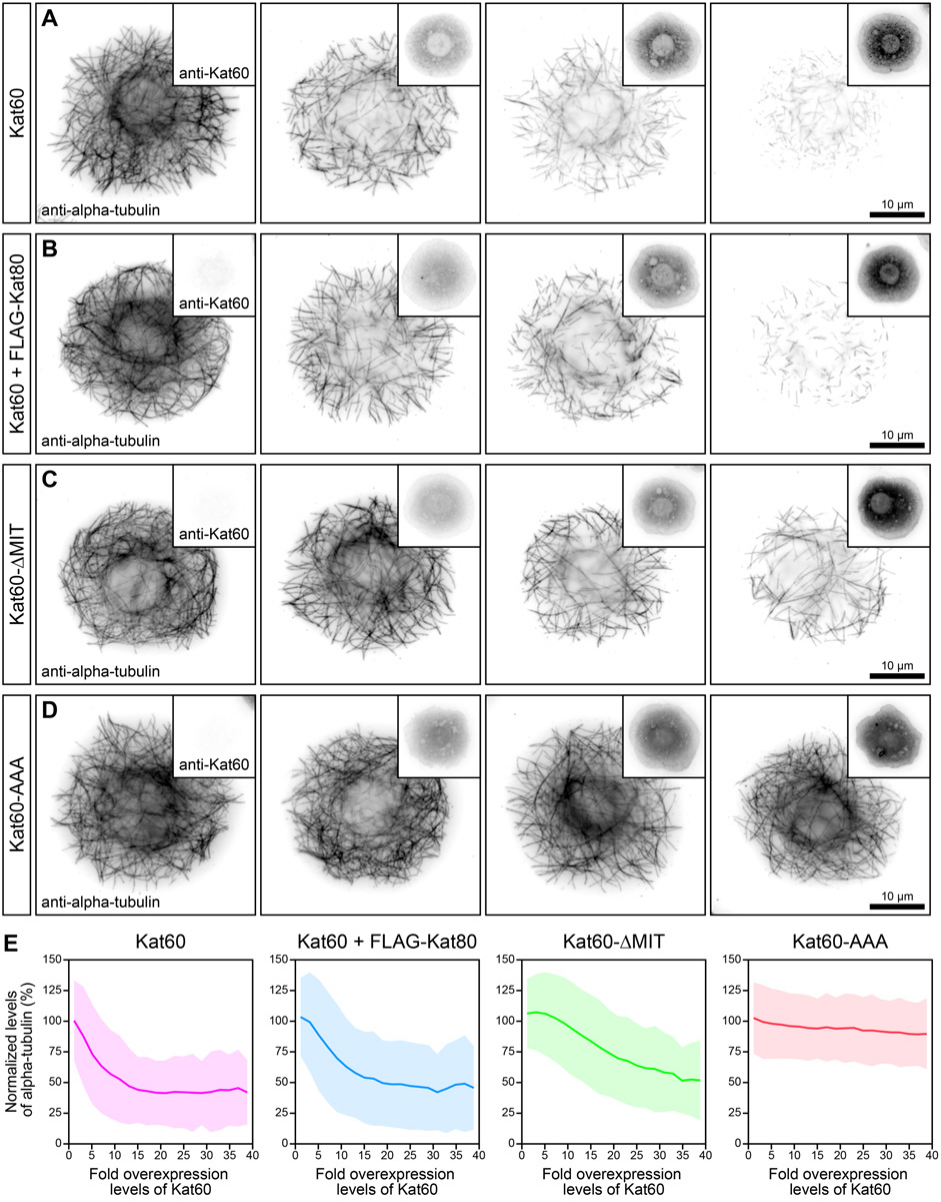
Kat60 lacking the MIT domain does not disassemble microtubules at low levels of accumulation in cells. (A-D) Immunofluorescence microscopy images of *Drosophila* S2 cells stably expressing GFP and copper-inducible Kat60 (A), Kat60 and FLAG-Kat80 (B), Kat60-ΔMIT (C), or Kat60-AAA (D) that were treated with both Kat60 and Kat80 UTR dsRNA for 7 days total. The cells described in A-D were also treated with 0–1.0 (A), 0–1.0 (B), 0–0.01 (C), or 0–0.01 mM CuSO_4_ (D) for 20 hours and immunostained for alpha-tubulin and Kat60. Alpha-tubulin and Kat60 images in each panel are displayed with the same scaling. (E) Line graphs of normalized levels of alpha-tubulin as a function of fold overexpression levels of Kat60 for the cells described in A-D. Normalized levels of alpha-tubulin are expressed as a percentage of the mean levels of alpha-tubulin in cells with fold overexpression levels of Kat60 below 0. Fold overexpression levels of Kat60 are expressed as a fraction of the difference in the mean levels of Kat60 between cells stably expressing GFP alone that were treated with control and both Kat60 and Kat80 UTR dsRNA for 7 days total. Data represent mean values ± standard deviation from cells with fold overexpression levels of Kat60 between 0 and 40, pooled from six independent experiments (see S3 Table for summary statistics of the single-cell measurements collected).

To circumvent the limitations highlighted above and reassess the contribution of the non-catalytic domains of *Drosophila* katanin to its microtubule-disassembly activity, we first expressed Kat60 alone or together with FLAG-Kat80, Kat60-ΔMIT, or Kat60-AAA over a broad range of levels in cells. We then used immunofluorescence microscopy and our single-cell assay to carefully examine the effects on microtubules as a function of the levels of these proteins. Cells expressing Kat60 together with FLAG-Kat80 exhibited microtubule disassembly in a concentration-dependent manner analogous to cells expressing Kat60 alone (Fig 6A and 6B). Likewise, these cells had reduced levels of alpha-tubulin similar to cells expressing full-length Kat60 alone (Fig 6E and S3 Table), suggesting that Kat80 does not affect the ability of Kat60 to disassemble microtubules. By contrast, cells expressing Kat60-ΔMIT at low levels did not display noticeably disassembled microtubules, although cells expressing Kat60-ΔMIT at high levels showed partially disassembled microtubules similar to cells expressing Kat60 at low levels (Fig 6A and 6C). Accordingly, the former cells did not have notably reduced levels of alpha-tubulin, whereas the latter cells had equivalent levels of alpha-tubulin compared to cells expressing Kat60 at low levels (Fig 6E and S3 Table). Thus, in addition to providing strong evidence that the MIT domain of Kat60 is important for its ability to disassemble microtubules at low levels of accumulation, these data further underscore the unique concentration-dependent contribution of the MIT domain of Kat60 to its activity. As predicted from our previous observations, cells expressing Kat60-AAA did not exhibit microtubule disassembly or have reduced levels of alpha-tubulin like cells expressing full-length Kat60 (Fig 6A, 6D, and 6E and S3 Table), strongly suggesting that the MIT domain and linker region of Kat60 cooperate to augment its microtubule-disassembly activity.

To verify that Kat60 alone or in the presence of FLAG-Kat80, Kat60-ΔMIT, and Kat60-AAA all required ATP to disassemble microtubules, we first expressed each of these proteins harboring a well-characterized mutation in a critical Walker A residue (K339A) designed to prevent ATP binding in cells. We then used our assay to measure the loss of microtubules in cells expressing each of these ATP-binding deficient mutants of Kat60 over an identical range of levels. As expected, cells expressing Kat60-K339A alone or together with FLAG-Kat80, Kat60-ΔMIT-K339A, or Kat60-AAA-K339A did not have reduced levels of alpha-tubulin compared to control cells (S4 Fig), demonstrating that ATP-binding deficient mutants of Kat60 do not disassemble microtubules.

### The MIT domain and linker region of Kat60 are required for its association with microtubules in cells

Given our findings that both Kat60-ΔMIT and Kat60-AAA do not disassemble microtubules like Kat60 in cells, we next investigated how the non-catalytic domains of *Drosophila* katanin regulate its microtubule-disassembly activity. Because the non-AAA region of katanin catalytic subunits from several species has been to shown to bind microtubules in vitro [16–19] and because a putative human katanin regulatory subunit has also been shown to bind microtubules in vitro [16], we reasoned that the non-catalytic domains of *Drosophila* katanin regulate its microtubule-disassembly activity by affecting its association with microtubules. In order to test this hypothesis, we developed a fluorescence microscopy assay to visualize the colocalization of Kat60 with microtubules in living cells. For this assay, we generated cells that stably express 1) RFP-tagged alpha-tubulin (RFP-alpha-tubulin) under the control of its own endogenous promoter and 2) GFP-tagged versions of Kat60 harboring the K339A mutation, either alone or together with Myc-Kat80, under the control of a copper-inducible promoter. We chose to examine the colocalization of ATP-binding deficient mutants of Kat60 with microtubules in this assay because they do not possess microtubule-disassembly activity and because an equivalent mutant of the human katanin catalytic subunit KATNA1 has been shown to possess microtubule-binding activity [15]. In this assay, we first induce cells with CuSO_4_ and then we acquire snapshot images of cells using total internal reflection fluorescence (TIRF) microscopy.

**Fig 7:**
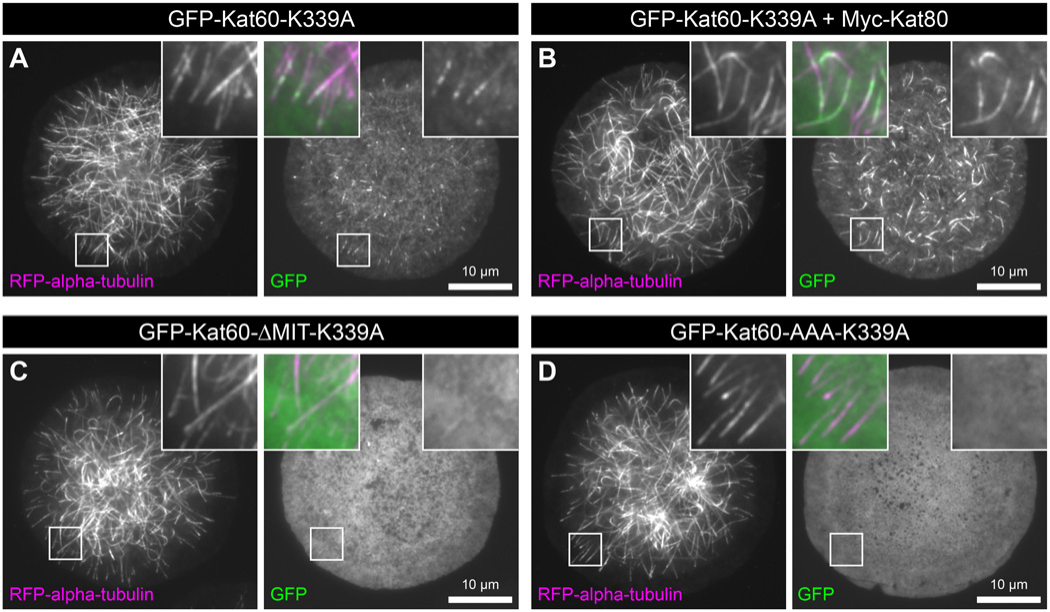
Kat60 lacking the MIT domain or the MIT domain and linker region detectably colocalizes with only a few, if any, microtubules in cells. (A-D) TIRF microscopy images of living *Drosophila* S2 cells stably expressing RFP-alpha-tubulin and copper-inducible GFP-Kat60-K339A (A), GFP-Kat60-K339A and Myc-Kat80 (B), GFP-Kat60-ΔMIT-K339A (C), or GFP-Kat60-AAA-K339A (D) that were treated with both Kat60 and Kat80 UTR dsRNA for 7 days total. The cells described in A-D were also treated with 0.1 (A), 0.1 (B), 0.1 (C), or 0.01 mM CuSO_4_ (D) for 20 hours. GFP images are displayed with the same scaling.

To test the hypothesis that the non-catalytic domains of *Drosophila* katanin affect its association with microtubules, we first expressed GFP-Kat60-K339A alone or together with Myc-Kat80, GFP-Kat60-ΔMIT-K339A, or GFP-Kat60-AAA-K339A in cells. We then used our live-cell assay to examine the colocalization of each of these proteins with microtubules. GFP-Kat60-K339A colocalized with the majority of microtubules in cells and it exhibited a discontinuous and punctate localization pattern on these microtubules (Fig 7A). Interestingly, GFP-Kat60-K339A in the presence of Myc-Kat80 also colocalized with the majority of microtubules in cells, however it exhibited a continuous localization pattern on these microtubules (Fig 7B), suggesting that Kat80 alters the association of Kat60 with microtubules. In contrast, GFP-Kat60-ΔMIT-K339A and GFP-Kat60-AAA-K339A detectably colocalized with only a few, if any, microtubules in cells (Fig 7C and 7D), providing strong evidence that the MIT domain and linker region of Kat60 are required for its association with microtubules. From these results, we conclude that the MIT domain and linker region of Kat60 augment its microtubule-disassembly activity by enhancing its association with microtubules.

### Model for regulation of *Drosophila* katanin by its non-catalytic domains

**Fig 8:**
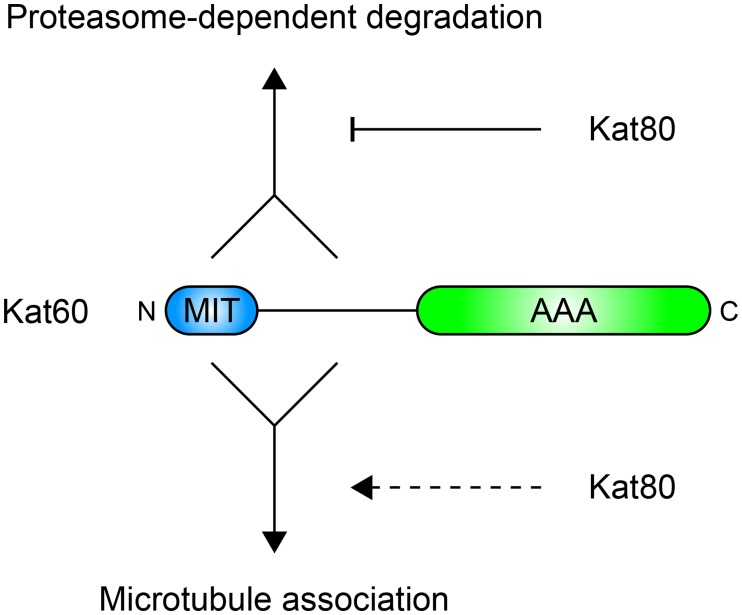
Model for regulation of *Drosophila* katanin by its non-catalytic domains. Schematic representation of the proposed contributions of the non-catalytic domains of *Drosophila* katanin to its proteasome-dependent degradation and microtubule association. Converging solid lines indicate cooperation between the MIT domain and linker region of Kat60 and solid lines with arrowheads indicate enhancement of the proteasome-dependent degradation and microtubule association of Kat60 by its MIT domain and linker region. The solid line with a blunt arrowhead indicates reduction of the proteasome-dependent degradation of Kat60 by Kat80 and the dashed line with an arrowhead indicates alteration of the microtubule association of Kat60 by Kat80. See text for details.

Collectively, our data suggest a model for how the non-catalytic domains of *Drosophila* katanin regulate microtubule severing (Fig 8). First, we speculate that the MIT domain of Kat60 physically interacts with Kat80—a well-established mode of interaction between katanin catalytic and regulatory subunits from several species [15,16]—and that the primary function of this interaction is to regulate the abundance of Kat60 itself. Our data and those of others [34,38,39] are consistent with a mechanism in which the MIT domain and linker region of Kat60 enhance its proteasome-dependent degradation by promoting interactions with ubiquitin ligase complexes, whereas Kat80 reduces the proteasome-dependent degradation of Kat60 by antagonizing such interactions. This mechanism could serve to maintain stoichiometric levels of Kat60 and Kat80 to ensure proper heterodimeric complex formation. In addition to regulating the abundance of Kat60, our data also demonstrate that the MIT domain and linker region of Kat60 regulate its microtubule-disassembly activity. In support of the katanin assembly model proposed by Hartman and Vale [17], our data suggest that the MIT domain and linker region of Kat60 enhance its initial targeting to microtubules. We speculate that the MIT domain and linker region of Kat60 also influence the subsequent balancing between Kat60-microtubule and Kat60-Kat60 interactions, however our data indicate that the MIT domain cannot be required for these interactions. On the other hand, our data suggest that Kat80 does not affect the microtubule-disassembly activity of Kat60 despite altering the association of Kat60 with microtubules. Previous studies of katanin from several species have shown that the katanin regulatory subunit potently stimulates the microtubule-severing activity of the katanin catalytic subunit in vitro [25] and that the heterodimeric complex exhibits distinct microtubule-binding properties in vitro [40]. Thus, we speculate Kat80 might function to target Kat60 to unique structural features or post-translational modifications of microtubules where it requires activation via some regulatory input to stimulate microtubule severing. Future work will focus on determining the functional importance of the microtubule-binding activity of Kat80 in living cells.

## Materials and Methods

### Plasmid construction

Multi-expression plasmids were constructed in the pMT/V5-His A expression plasmid backbone (Invitrogen) and additional expression cassettes were cloned into unique restriction enzyme sites engineered into regions outside of the copper-inducible expression cassette. Constitutive expression cassettes were obtained by PCR amplification of the pIZ/V5-His expression plasmid (Invitrogen) containing the CDS of GFP, the pCoBlast expression plasmid containing the CDS of the blasticidin-resistance protein (Invitrogen), or the pCoHygro expression plasmid containing the CDS of the hygromycin-resistance protein (Invitrogen). The *Drosophila* alpha-tubulin expression cassette was created by PCR amplification of *Drosophila* S2 cell genomic DNA containing the 5’ and 3’ UTR of alpha-tubulin and expression plasmids containing the CDS of RFP [41] and alpha-tubulin [42] and fusion of the resulting PCR products using the In-Fusion HD cloning kit (Clontech Laboratories, Inc.). All copper-inducible transgenes were created by cloning PCR-amplified CDS into the multiple cloning site of the copper-inducible expression cassette. Transgenes encoding untagged full-length and deletion mutants of *Drosophila* Kat60 were created by PCR amplification of the CDS of full-length Kat60 (amino acids 1–572), Kat60-ΔMIT (amino acids 97–572), or Kat60-AAA (amino acids 265–572) from the Berkeley *Drosophila* Genome Project (BDGP) cDNA clone RE17942 (*Drosophila* Genomics Resource Center). The transgene encoding internally GFP-tagged full-length Kat60 was created by PCR amplification of the CDS of Kat60-MIT (amino acids 1–96), GFP, and Kat60-ΔMIT and fusion of the resulting PCR products using the In-Fusion HD cloning kit (Clontech Laboratories, Inc.). Transgenes encoding NH_2_-terminally GFP-tagged deletion mutants of Kat60 were created by PCR amplification of the CDS of GFP and Kat60-ΔMIT or Kat60-AAA and fusion of the resulting PCR products using the In-Fusion HD cloning kit (Clontech Laboratories, Inc.). Transgenes encoding NH_2_-terminally epitope-tagged full-length *Drosophila* Kat80 were created by PCR amplification of the CDS of full-length Kat80 (amino acids 1–819) from the BDGP cDNA clone LD44201 (*Drosophila* Genomics Resource Center) with the CDS of Myc or FLAG engineered onto the 5’ end of the PCR product. All point mutations were introduced by a PCR-based site-directed mutagenesis method and all plasmids were fully sequenced. The multi-expression plasmids constructed in this study are shown in S4 Table.

### Cell culture and generation of stable cell lines

*Drosophila* S2 cells were maintained in a room temperature (RT) incubator in S2 cell medium (S2M: Schneider’s *Drosophila* medium (Invitrogen) supplemented with 10% heat-inactivated FBS (Invitrogen) and 1x antibiotic-antimycotic solution (Invitrogen)). For generation of stable cell lines, cells were transiently transfected with the multi-expression plasmids described in S4 Table using the FuGENE HD transfection reagent (Promega) according to the manufacturer’s instructions. After transfection, cells were treated with 50 μg/mL blasticidin (Invitrogen), either alone or in combination, with 500 μg/mL hygromycin (Invitrogen) every 3 days for 4 weeks total. The stable cell lines generated in this study are shown in S5 Table.

### RNAi and inducible protein expression

DNA templates for dsRNA synthesis were obtained by PCR amplification of the pFastBacHT-CAT expression plasmid (Invitrogen), BDGP cDNA clones, or S2 cell genomic DNA using the gene-specific primer sequences shown in S6 Table. dsRNA was synthesized using the T7 RiboMAX large scale RNA production system (Promega) according to the manufacturer’s instructions. For RNAi, cells were treated with 20 μg/mL dsRNA every 2 days for 7 days total. For inducible protein expression, cells were treated with 0.01–1.0 mM CuSO_4_ for 16–20 hours.

### Pulsed-induction of protein expression and drug treatment

Cells were treated with 0.01–1.0 mM CuSO_4_ for 16 hours, washed thrice with S2M, and treated with DMSO (Sigma-Aldrich) or 50 μM MG132 (Sigma-Aldrich) for 0–12 hours.

### Antibody production

6xHis- and GST-tagged Kat60-AAA proteins were bacterially expressed and purified using Ni-NTA agarose (Qiagen) or glutathione sepharose (GE Healthcare). Polyclonal antibodies were generated against the 6xHis-tagged purified protein (Pocono Rabbit Farm and Laboratory) and affinity purified from sera using the GST-tagged purified protein coupled to CNBr-activated sepharose (GE Healthcare).

### Immunoblotting

Protein samples were resolved by SDS-PAGE and transferred to nitrocellulose membranes (Whatman). Membranes were blocked with membrane blocking buffer (MBB: 5% non-fat milk (LabScientific) in PBS containing 0.1% Tween-20) for 1 hour at RT. For chemiluminescent immunodetection of proteins, membranes were first incubated with rabbit anti-Kat60 (0.1–1 μg/mL) or mouse anti-FLAG (2 μg/mL; clone M2; Sigma-Aldrich) antibodies in MBB for 24 hours at 4°C and then incubated with HRP-conjugated goat anti-rabbit IgG (0.1–1 μg/mL; Sigma-Aldrich) or HRP-conjugated goat anti-mouse IgG (2 μg/mL; Sigma-Aldrich) antibodies in MBB for 1 hour at RT. Protein bands were visualized by incubating the membranes with enhanced chemiluminescent substrate (Pierce) and exposing the membranes to autoradiography film (GeneMate). For fluorescent immunodetection of proteins, membranes were first incubated with mouse anti-actin (1:10,000; clone C4; Millipore) or mouse anti-GFP (0.1 μg/mL; clone JL-8; Clontech Laboratories, Inc.) antibodies in MBB for 24 hours at 4°C and then incubated with Cy3-conjugated goat anti-mouse IgG (0.02 μg/mL; Jackson ImmunoResearch Laboratories, Inc.) antibodies in MBB for 1 hour at RT. Protein bands were visualized by fluorescence scanning of the membranes using a variable mode imager (Typhoon Trio; GE Healthcare).

### Cell plating

Cells were plated at a density of 6 x 10^4^ cells per well into 96-well glass-bottom microplates (0.17 mm glass; Matrical Bioscience) pre-washed with alcoholic potassium hydroxide and pre-coated with 0.5 mg/mL concanavalin A (MP Biomedicals). After plating, cells were washed with S2M prior to immunostaining or imaging in fresh S2M.

### Immunostaining

Cells were washed with cell fixation buffer (CFB: 100 mM PIPES, pH 6.8, 1 mM EGTA, and 1 mM MgCL_2_) and fixed with 10% paraformaldehyde (Electron Microscopy Sciences) in CFB for 10 minutes. Cells were permeabilized by washing with PBS containing 0.1% Triton X-100 (PBST) and blocked by incubating with cell blocking buffer (CBB: 5% normal goat serum (Sigma-Aldrich) in PBST) for 10 minutes at RT. For immunostaining, cells were first incubated with rabbit anti-Kat60 (1 μg/mL), rabbit anti-Myc (1 μg/mL; Sigma-Aldrich), or mouse anti-alpha-tubulin (10 μg/mL; clone DM1A; Sigma-Aldrich) antibodies in CBB for 24 hours at 4°C and then incubated with Cy5-conjugated goat anti-rabbit IgG (1 μg/mL; Jackson ImmunoResearch, Inc.) or Cy3-conjugated goat anti-mouse IgG (2 μg/mL; Jackson ImmunoResearch, Inc.) antibodies in CBB for 2 hours at RT. After immunostaining, cells were incubated with 10 μg/mL Hoechst 33342 (Invitrogen) in PBST for 10 minutes at RT and cells were mounted in fluorescence mounting medium (Dako).

### Imaging

Images were acquired using an inverted microscope (Eclipse Ti-E; Nikon) equipped with a 20x 0.75 NA Plan Apochromat (Nikon) or 100x 1.49 NA TIRF Plan Apochromat objective (Nikon), a cooled charge-coupled device camera (Clara; Andor), a mercury lamp illumination system for epi-fluorescence (Intensilight; Nikon), a motorized shutter for epi-fluorescence (Ludl Electronic Products Ltd.), a laser unit (Nikon) housing 488 nm and 561 nm solid-state lasers (Sapphire LP; Coherent, Inc.), a TI-LUSU shutter unit for controlling motorized shutters in the laser unit (Nikon), a motorized laser TIRF illumination unit (Nikon), a motorized fluorescence filter cube rotating turret (Nikon), single-band fluorescence filter sets for Hoechst 33342 (exciter (EX): 360/20x, beam splitter (BS): 400, emitter (EM): 460/15m; Nikon), GFP (EX: ZET488/10x, BS: ZT488rdc, EM: ET525/50m + HHQ500lp; Chroma Technology Corporation), RFP/Cy3 (EX: ZET561/10x, BS: ZT561rdc, EM: ET600/50m + HHQ575lp; Chroma Technology Corporation), and Cy5 (EX: ZET635/20x, BS: ZT640rdc, EM: ET655lp + HHQ660lp; Chroma Technology Corporation), a motorized XY stage (Nikon), and a motorized nosepiece combined with the Perfect Focus System for Z-axis control (Nikon). NIS-Elements AR software (Nikon) was used to control the imaging system and images were acquired using identical system settings (e.g. exposure times, camera bit depth, binning, and gain) for each well of the same 96-well glass-bottom microplate.

### Image processing and analysis

High-content immunofluorescence microscopy image processing and analysis was performed using the open-source CellProfiler software [32]. In brief, images were illumination corrected and background subtracted prior to identification of objects and measurement of immunofluorescence intensities. For identification of nuclei, the DNA images were segmented using the Otsu Global two-class thresholding method and the identified nuclei were used to seed the identification of individual cells. For identification of individual cells, the GFP images were segmented using the Watershed—Image method and the Otsu Global three-class thresholding method and the identified cells were used to create cell outlines. For single-cell measurement of alpha-tubulin and Kat60 immunofluorescence intensities, the average pixel intensities were extracted from each outlined cell based on the alpha-tubulin and Kat60 images, respectively.

## Supporting Information

S1 Fig Development of a single-cell assay to measure the microtubule-disassembly activity of Kat60 using automated microscopy. (A-D) Schematic representation (Left) and example high-content immunofluorescence microscopy images (Middle and Right) of *Drosophila* S2 cells stably expressing GFP and copper-inducible Kat60 that were treated with both Kat60 and Kat80 UTR dsRNA for 7 days total. The cells were also treated with 1.0 mM CuSO_4_ for 20 hours and stained for DNA and immunostained for alpha-tubulin and Kat60. (A) Original DNA image (Middle) and image of nuclei identified from the DNA image created by CellProfiler software (Right). (B) Original GFP image (Middle) and image of individual cells identified from the GFP image created by CellProfiler software (Right). (C) Original alpha-tubulin image (Middle) and alpha-tubulin image with cell outlines created by CellProfiler software (Right). (D) Original Kat60 image (Middle) and Kat60 image with cell outlines created by CellProfiler software (Right).
(TIF)

S2 Fig Both Kat60 and Kat80 UTR dsRNA treatments reduce steady-state Kat60 levels in cells. Immunoblots of *Drosophila* S2 cell lysates prepared from cells stably expressing GFP alone that were treated with control (lane 1), Kat60 UTR (lane 2), Kat80 UTR (lane 3), or both Kat60 and Kat80 UTR dsRNA (lane 4) for 7 days total. Molecular weights (in Kd) are shown on the left.
(TIF)

S3 Fig Depletion of Kat60 and Kat80 causes minimal perturbation of steady-state alpha-tubulin levels in cells. Histograms of normalized levels of alpha-tubulin (Left) and normalized levels of Kat60 (Right) in *Drosophila* S2 cells stably expressing GFP alone that were treated with control (black, 32,389 total cells) or both Kat60 and Kat80 UTR dsRNA (gray, 32,142 total cells) for 7 days total. Normalized levels of alpha-tubulin and Kat60 are expressed as a percentage of the mean levels of alpha-tubulin and Kat60, respectively, in cells stably expressing GFP alone that were treated with control dsRNA for 7 days total. Data are pooled from three independent experiments.
(TIF)

S4 Fig ATP-binding deficient mutants of Kat60 do not disassemble microtubules in cells. Bar graph of normalized levels of alpha-tubulin in *Drosophila* S2 cells stably expressing GFP and copper-inducible Kat60-K339A (magenta, 11,938 total cells), Kat60-K339A and FLAG-Kat80 (blue, 7,956 total cells), Kat60-ΔMIT-K339A (green, 9,494 total cells), or Kat60-AAA-K339A (red, 4,240 total cells) that were treated with both Kat60 and Kat80 UTR dsRNA for 7 days total. The cells were also treated with 0–1.0 (magenta), 0–1.0 (blue), 0–0.01 (green), or 0–0.01 mM CuSO_4_ (red) for 20 hours. Normalized levels of alpha-tubulin are expressed as a percentage of the mean levels of alpha-tubulin in cells stably expressing GFP alone that were treated with both Kat60 and Kat80 UTR dsRNA for 7 days total. Fold overexpression levels of Kat60 are expressed as a fraction of the difference in the mean levels of Kat60 between cells stably expressing GFP alone that were treated with control and both Kat60 and Kat80 UTR dsRNA for 7 days total. Data represent mean values ± standard deviation from cells with fold overexpression levels of Kat60 between 0 and 40, pooled from three independent experiments.
(TIF)

S1 Table Summary statistics of the single-cell measurements shown in Fig 3. Normalized levels of alpha-tubulin and fold overexpression levels of Kat60 in *Drosophila* S2 cells stably expressing GFP and copper-inducible Kat60 (rows 1–4) or FLAG-Kat80 (rows 5–8) that were treated with both Kat60 and Kat80 UTR dsRNA for 7 days total. The cells were also treated with 0 (rows 1 and 5), 0.01 (rows 2 and 6), 0.1 (rows 3 and 7), or 1.0 mM CuSO_4_ (rows 4 and 8) for 20 hours and immunostained for alpha-tubulin and Kat60. Normalized levels of alpha-tubulin are expressed as a percentage of the mean levels of alpha-tubulin in cells stably expressing GFP alone that were treated with both Kat60 and Kat80 UTR dsRNA for 7 days total. Fold overexpression levels of Kat60 are expressed as a fraction of the difference in the mean levels of Kat60 between cells stably expressing GFP alone that were treated with control and both Kat60 and Kat80 UTR dsRNA for 7 days total. Data are pooled from three independent experiments. The following abbreviations are used: total, total cell number; SD, standard deviation; and IQR, interquartile range.
(TIF)

S2 Table Summary statistics of the single-cell measurements shown in Fig 4. Normalized levels of alpha-tubulin and fold overexpression levels of Kat60 in *Drosophila* S2 cells stably expressing GFP and copper-inducible Kat60 (rows 1–4), Kat60 and FLAG-Kat80 (rows 5–8), Kat60-ΔMIT (rows 9–12), or Kat60-AAA (rows 13–16) that were treated with both Kat60 and Kat80 UTR dsRNA for 7 days total. The cells described were also treated with 0 (rows 1, 5, 9, and 13), 0.01 (rows 2, 6, 10, and 14), 0.1 (rows 3, 7, 11, and 15), or 1.0 mM CuSO_4_ (rows 4, 8, 12, and 16) for 20 hours and immunostained for alpha-tubulin and Kat60. Normalized levels of alpha-tubulin are expressed as a percentage of the mean levels of alpha-tubulin in cells stably expressing GFP alone that were treated with both Kat60 and Kat80 UTR dsRNA for 7 days total. Fold overexpression levels of Kat60 are expressed as a fraction of the difference in the mean levels of Kat60 between cells stably expressing GFP alone that were treated with control and both Kat60 and Kat80 UTR dsRNA for 7 days total. Data are pooled from three independent experiments. The following abbreviations are used: total, total cell number; SD, standard deviation; and IQR, interquartile range.
(TIF)

S3 Table Summary statistics of the single-cell measurements shown in Fig 6. Normalized levels of alpha-tubulin in *Drosophila* S2 cells stably expressing GFP and copper-inducible Kat60 (column 1), Kat60 and FLAG-Kat80 (column 2), Kat60-ΔMIT (column 3), or Kat60-AAA (column 4) that were treated with both Kat60 and Kat80 UTR dsRNA for 7 days total. The cells were also treated with 0–1.0 (column 1), 0–1.0 (column 2), 0–0.01 (column 3), or 0–0.01 mM CuSO_4_ (column 4) for 20 hours and immunostained for alpha-tubulin and Kat60. Normalized levels of alpha-tubulin are expressed as a percentage of the mean levels of alpha-tubulin in cells with fold overexpression levels of Kat60 below 0. Fold overexpression levels of Kat60 are expressed as a fraction of the difference in the mean levels of Kat60 between cells stably expressing GFP alone that were treated with control and both Kat60 and Kat80 UTR dsRNA for 7 days total. Data are pooled from six independent experiments. The following abbreviations are used: total, total cell number; and SD, standard deviation.
(TIF)

S4 Table List of the multi-expression plasmids constructed in this study. Multi-expression plasmids are shown with individual expression cassettes separated by forward slashes and with CDS separated from promoters by double colons. The following abbreviations are used: MT, copper-inducible metallothionein promoter; OP, constitutive OpIE2 promoter. CO, constitutive copia promoter; AT, endogenous alpha-tubulin promoter; Blast, blasticidin-resistance protein; and Hygro, hygromycin-resistance protein.
(TIF)

S5 Table List of the *Drosophila* S2 cell lines generated in this study. ^a^Two independent *Drosophila* S2 cell lines stably expressing GFP and copper-inducible Kat60 were generated and used in this study. The first was used to acquire the data shown in S1 Fig and S3 Fig and the second was used to acquire the data shown in Fig 4, Fig 5, and Fig 6.
(TIF)

S6 Table List of the DNA templates and gene-specific primer sequences used in this study for dsRNA synthesis. ^a^Primer sequences are listed as 5’ to 3’ and each is preceded with the T7 promoter sequence 5’-TAATACGACTCACTATAGGG-3’.
(TIF)
