# Supplementary figures and images for: The Non-Catalytic Domains of Drosophila Katanin Regulate Its Abundance and Microtubule-Disassembly Activity
Source: PLoS One. 2015 Apr 17;10(4):e0123912. doi: 10.1371/journal.pone.0123912 (PMC4401518; doi:10.1371/journal.pone.0123912)

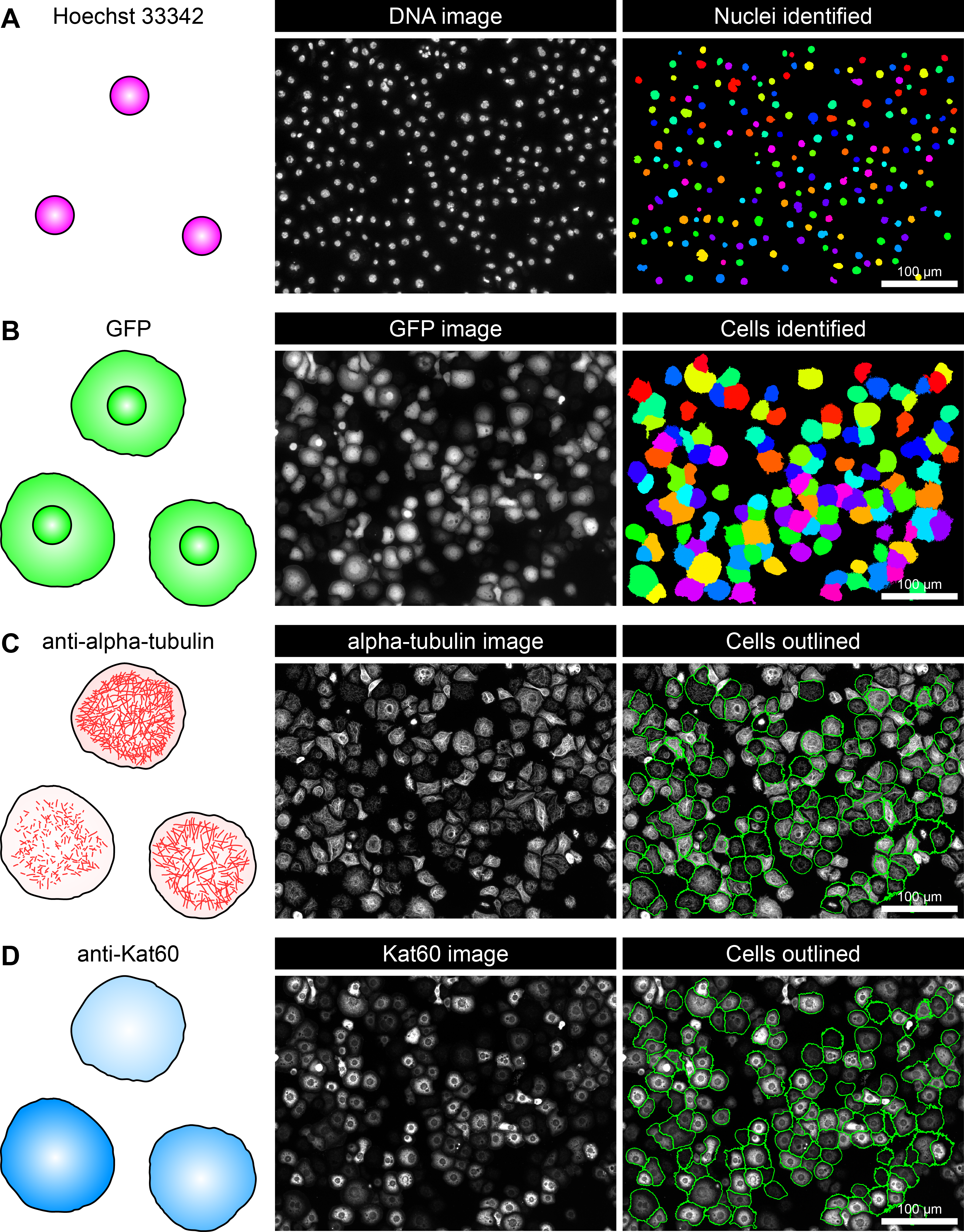

Supplement: S1 Fig — (A-D) Schematic representation (Left) and example high-content immunofluorescence microscopy images (Middle and Right) of Drosophila S2 cells stably expressing GFP and copper-inducible Kat60 that were treated with both Kat60 and Kat80 UTR dsRNA for 7 days total. The cells were also treated with 1.0 mM CuSO4 for 20 hours and stained for DNA and immunostained for alpha-tubulin and Kat60. (A) Original DNA image (Middle) and image of nuclei identified from the DNA image created by CellProfiler software (Right). (B) Original GFP image (Middle) and image of individual cells identified from the GFP image created by CellProfiler software (Right). (C) Original alpha-tubulin image (Middle) and alpha-tubulin image with cell outlines created by CellProfiler software (Right). (D) Original Kat60 image (Middle) and Kat60 image with cell outlines created by CellProfiler software (Right). (TIF) [file pone.0123912.s001.tif]

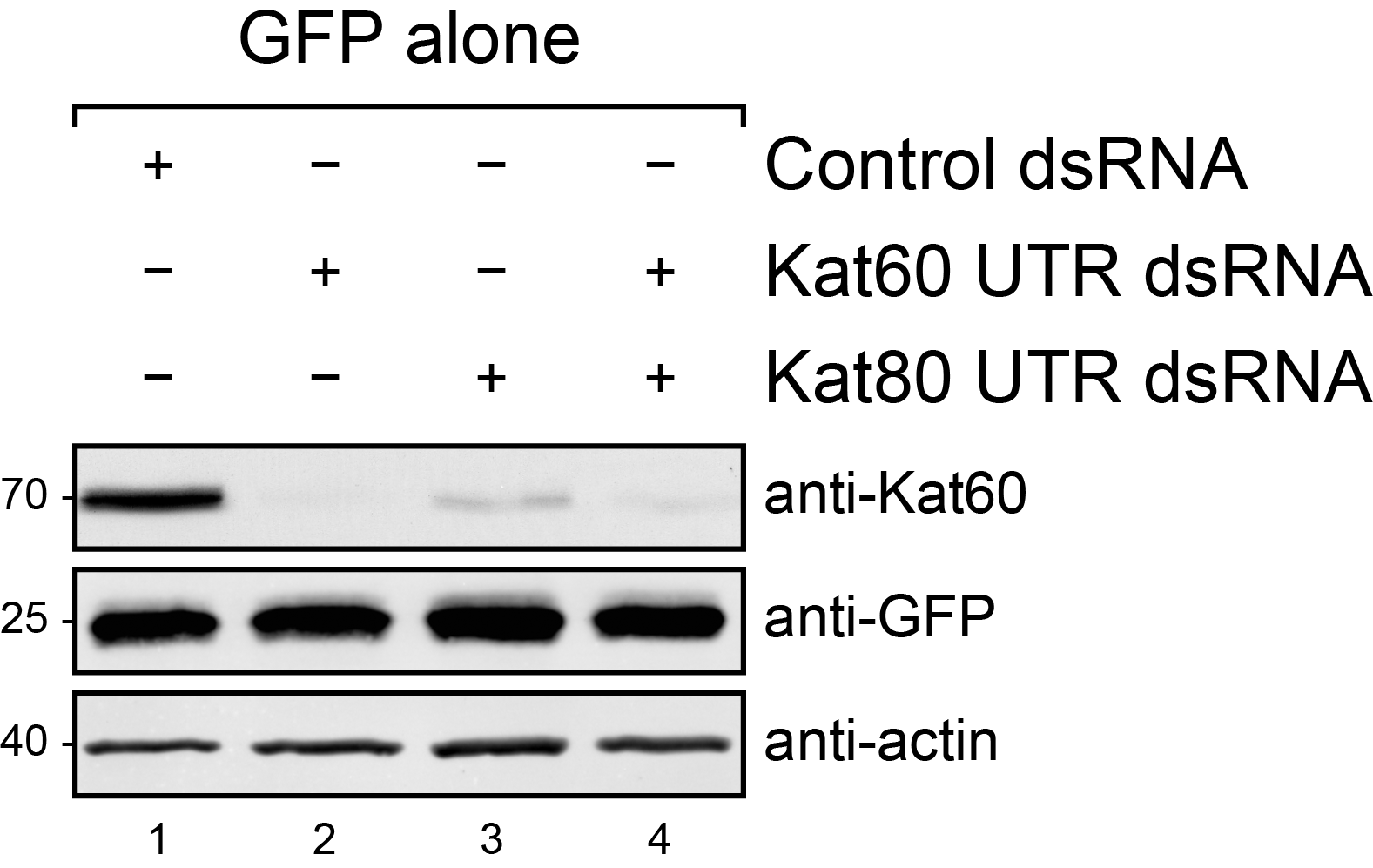

Supplement: S2 Fig — Immunoblots of Drosophila S2 cell lysates prepared from cells stably expressing GFP alone that were treated with control (lane 1), Kat60 UTR (lane 2), Kat80 UTR (lane 3), or both Kat60 and Kat80 UTR dsRNA (lane 4) for 7 days total. Molecular weights (in Kd) are shown on the left. (TIF) [file pone.0123912.s002.tif]

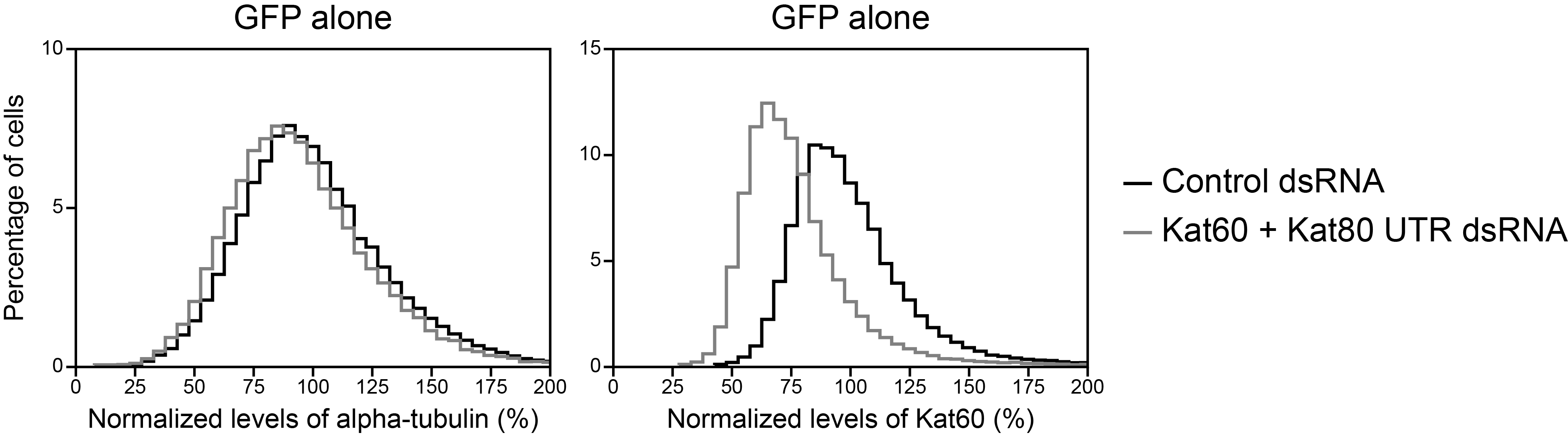

Supplement: S3 Fig — Histograms of normalized levels of alpha-tubulin (Left) and normalized levels of Kat60 (Right) in Drosophila S2 cells stably expressing GFP alone that were treated with control (black, 32,389 total cells) or both Kat60 and Kat80 UTR dsRNA (gray, 32,142 total cells) for 7 days total. Normalized levels of alpha-tubulin and Kat60 are expressed as a percentage of the mean levels of alpha-tubulin and Kat60, respectively, in cells stably expressing GFP alone that were treated with control dsRNA for 7 days total. Data are pooled from three independent experiments. (TIF) [file pone.0123912.s003.tif]

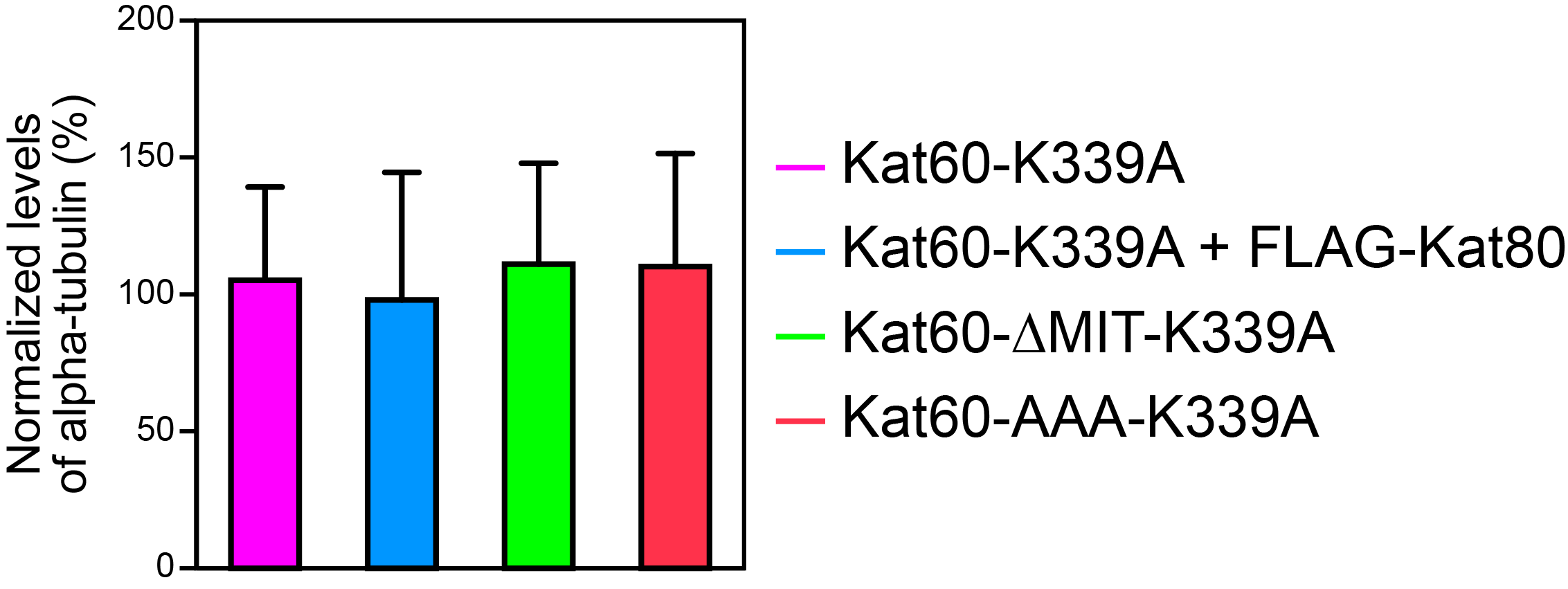

Supplement: S4 Fig — Bar graph of normalized levels of alpha-tubulin in Drosophila S2 cells stably expressing GFP and copper-inducible Kat60-K339A (magenta, 11,938 total cells), Kat60-K339A and FLAG-Kat80 (blue, 7,956 total cells), Kat60-ΔMIT-K339A (green, 9,494 total cells), or Kat60-AAA-K339A (red, 4,240 total cells) that were treated with both Kat60 and Kat80 UTR dsRNA for 7 days total. The cells were also treated with 0–1.0 (magenta), 0–1.0 (blue), 0–0.01 (green), or 0–0.01 mM CuSO4 (red) for 20 hours. Normalized levels of alpha-tubulin are expressed as a percentage of the mean levels of alpha-tubulin in cells stably expressing GFP alone that were treated with both Kat60 and Kat80 UTR dsRNA for 7 days total. Fold overexpression levels of Kat60 are expressed as a fraction of the difference in the mean levels of Kat60 between cells stably expressing GFP alone that were treated with control and both Kat60 and Kat80 UTR dsRNA for 7 days total. Data represent mean values ± standard deviation from cells with fold overexpression levels of Kat60 between 0 and 40, pooled from three independent experiments. (TIF) [file pone.0123912.s004.tif]

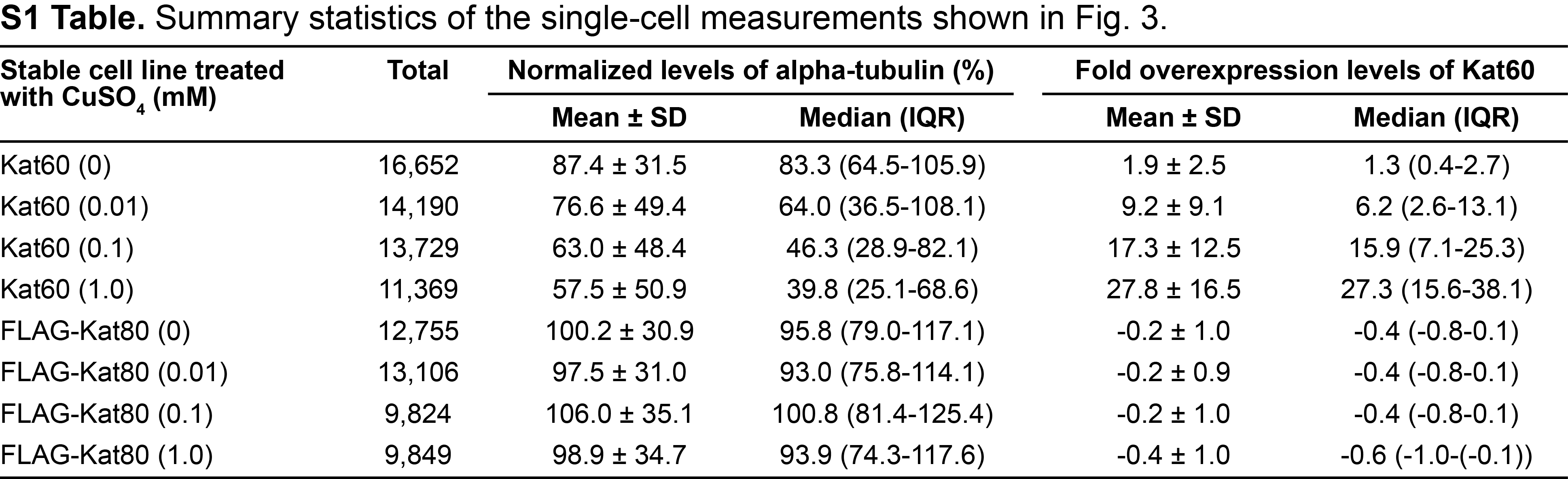

Supplement: S1 Table — Normalized levels of alpha-tubulin and fold overexpression levels of Kat60 in Drosophila S2 cells stably expressing GFP and copper-inducible Kat60 (rows 1–4) or FLAG-Kat80 (rows 5–8) that were treated with both Kat60 and Kat80 UTR dsRNA for 7 days total. The cells were also treated with 0 (rows 1 and 5), 0.01 (rows 2 and 6), 0.1 (rows 3 and 7), or 1.0 mM CuSO4 (rows 4 and 8) for 20 hours and immunostained for alpha-tubulin and Kat60. Normalized levels of alpha-tubulin are expressed as a percentage of the mean levels of alpha-tubulin in cells stably expressing GFP alone that were treated with both Kat60 and Kat80 UTR dsRNA for 7 days total. Fold overexpression levels of Kat60 are expressed as a fraction of the difference in the mean levels of Kat60 between cells stably expressing GFP alone that were treated with control and both Kat60 and Kat80 UTR dsRNA for 7 days total. Data are pooled from three independent experiments. The following abbreviations are used: total, total cell number; SD, standard deviation; and IQR, interquartile range. (TIF) [file pone.0123912.s005.tif]

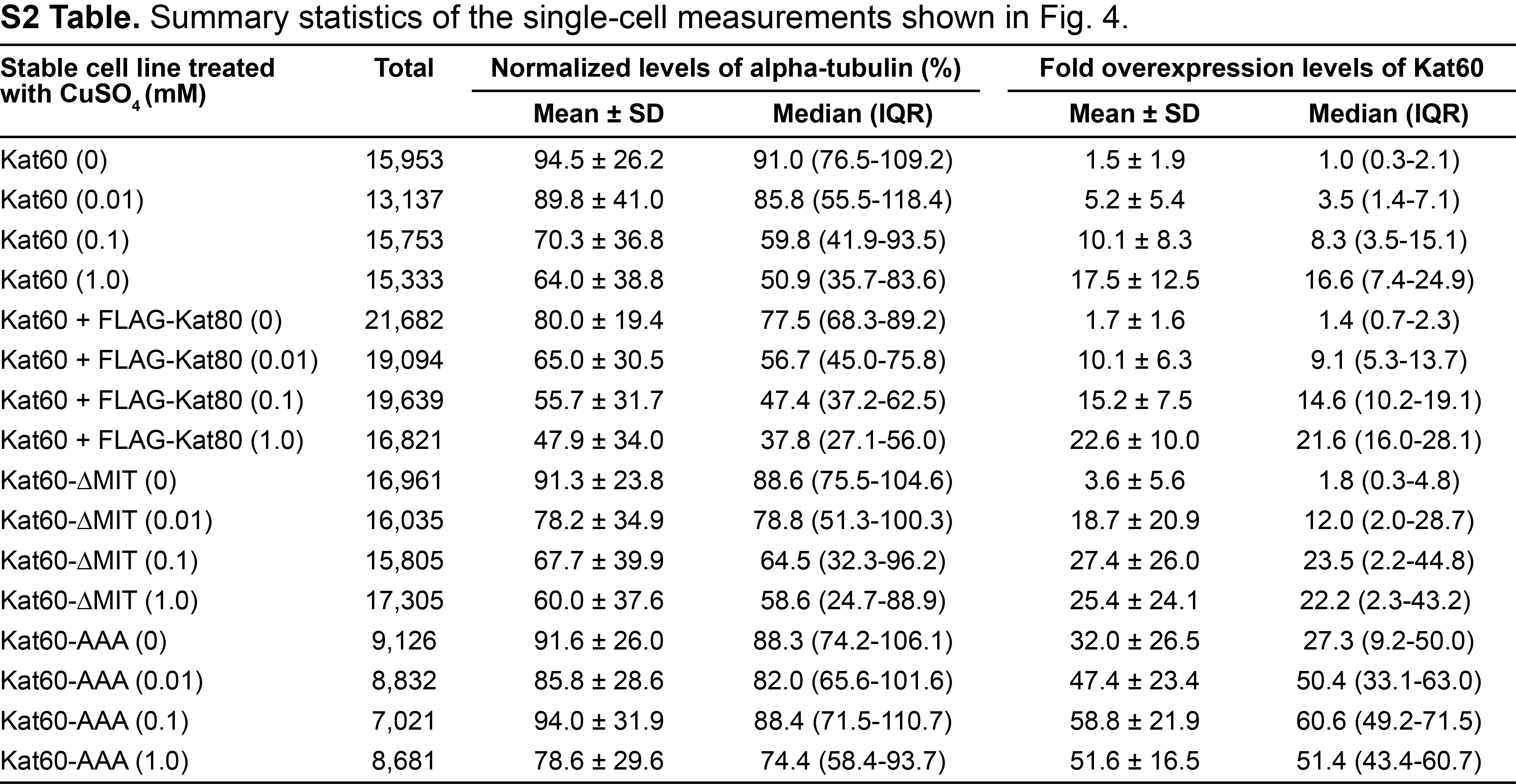

Supplement: S2 Table — Normalized levels of alpha-tubulin and fold overexpression levels of Kat60 in Drosophila S2 cells stably expressing GFP and copper-inducible Kat60 (rows 1–4), Kat60 and FLAG-Kat80 (rows 5–8), Kat60-ΔMIT (rows 9–12), or Kat60-AAA (rows 13–16) that were treated with both Kat60 and Kat80 UTR dsRNA for 7 days total. The cells described were also treated with 0 (rows 1, 5, 9, and 13), 0.01 (rows 2, 6, 10, and 14), 0.1 (rows 3, 7, 11, and 15), or 1.0 mM CuSO4 (rows 4, 8, 12, and 16) for 20 hours and immunostained for alpha-tubulin and Kat60. Normalized levels of alpha-tubulin are expressed as a percentage of the mean levels of alpha-tubulin in cells stably expressing GFP alone that were treated with both Kat60 and Kat80 UTR dsRNA for 7 days total. Fold overexpression levels of Kat60 are expressed as a fraction of the difference in the mean levels of Kat60 between cells stably expressing GFP alone that were treated with control and both Kat60 and Kat80 UTR dsRNA for 7 days total. Data are pooled from three independent experiments. The following abbreviations are used: total, total cell number; SD, standard deviation; and IQR, interquartile range. (TIF) [file pone.0123912.s006.tif]

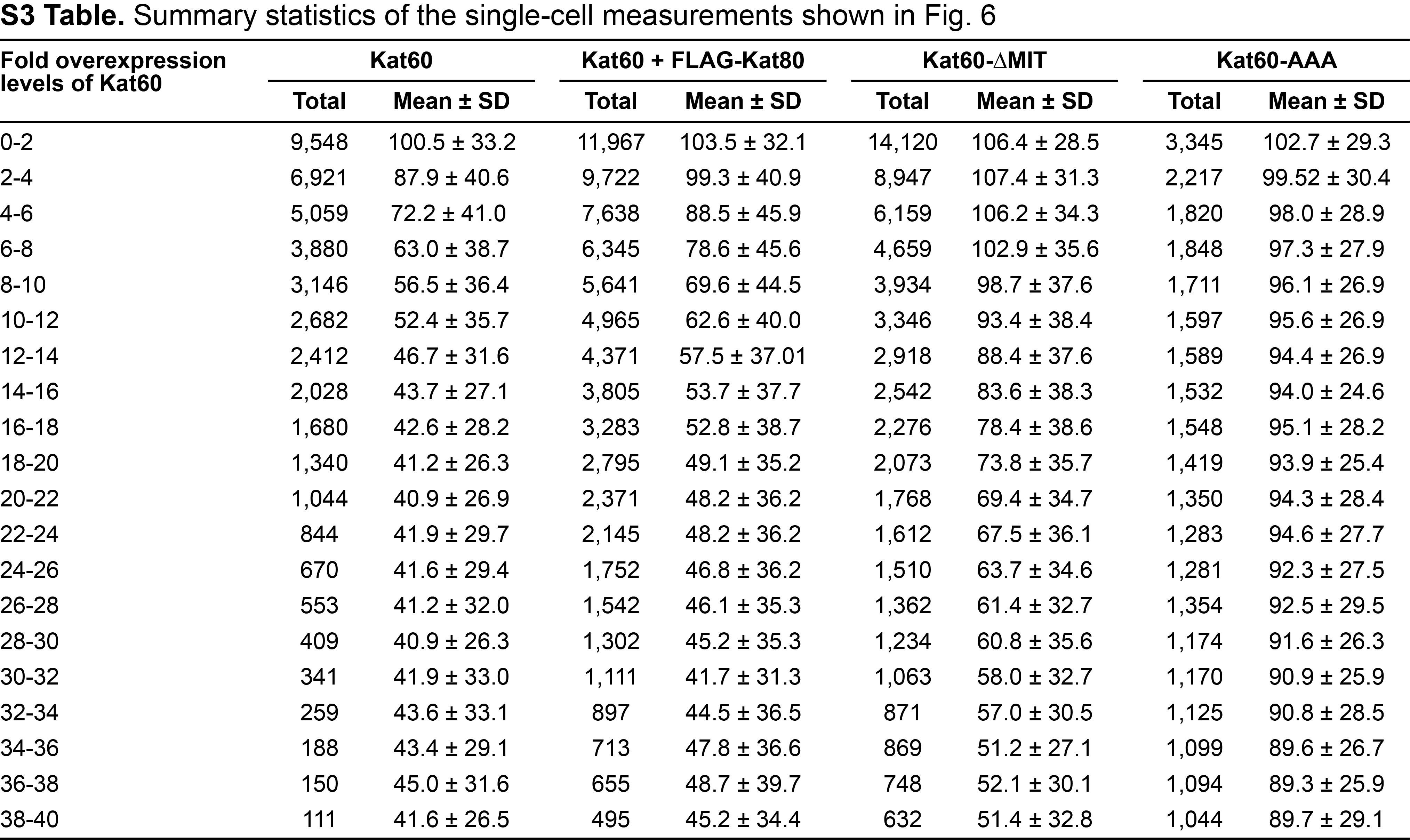

Supplement: S3 Table — Normalized levels of alpha-tubulin in Drosophila S2 cells stably expressing GFP and copper-inducible Kat60 (column 1), Kat60 and FLAG-Kat80 (column 2), Kat60-ΔMIT (column 3), or Kat60-AAA (column 4) that were treated with both Kat60 and Kat80 UTR dsRNA for 7 days total. The cells were also treated with 0–1.0 (column 1), 0–1.0 (column 2), 0–0.01 (column 3), or 0–0.01 mM CuSO4 (column 4) for 20 hours and immunostained for alpha-tubulin and Kat60. Normalized levels of alpha-tubulin are expressed as a percentage of the mean levels of alpha-tubulin in cells with fold overexpression levels of Kat60 below 0. Fold overexpression levels of Kat60 are expressed as a fraction of the difference in the mean levels of Kat60 between cells stably expressing GFP alone that were treated with control and both Kat60 and Kat80 UTR dsRNA for 7 days total. Data are pooled from six independent experiments. The following abbreviations are used: total, total cell number; and SD, standard deviation. (TIF) [file pone.0123912.s007.tif]

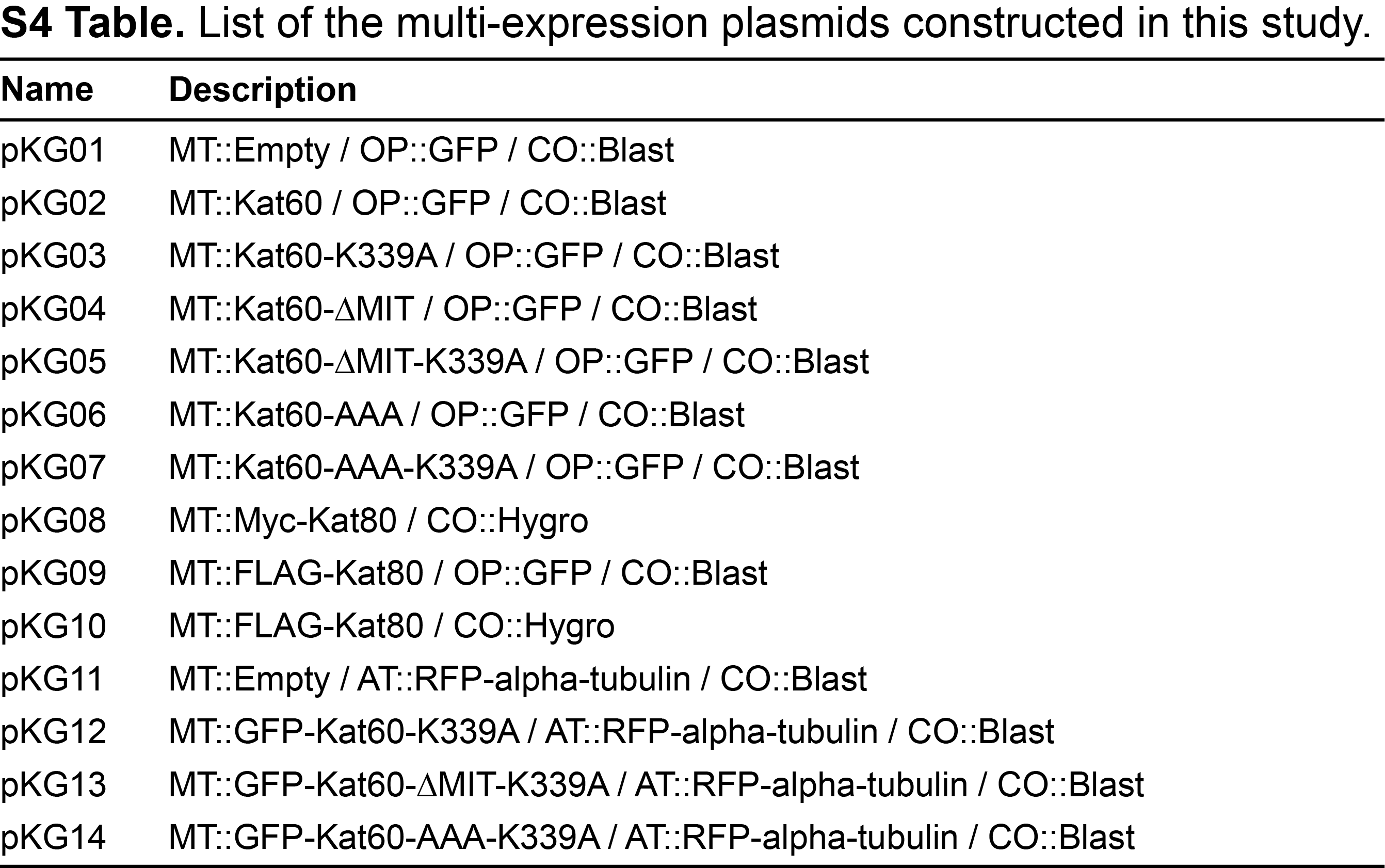

Supplement: S4 Table — Multi-expression plasmids are shown with individual expression cassettes separated by forward slashes and with CDS separated from promoters by double colons. The following abbreviations are used: MT, copper-inducible metallothionein promoter; OP, constitutive OpIE2 promoter. CO, constitutive copia promoter; AT, endogenous alpha-tubulin promoter; Blast, blasticidin-resistance protein; and Hygro, hygromycin-resistance protein. (TIF) [file pone.0123912.s008.tif]

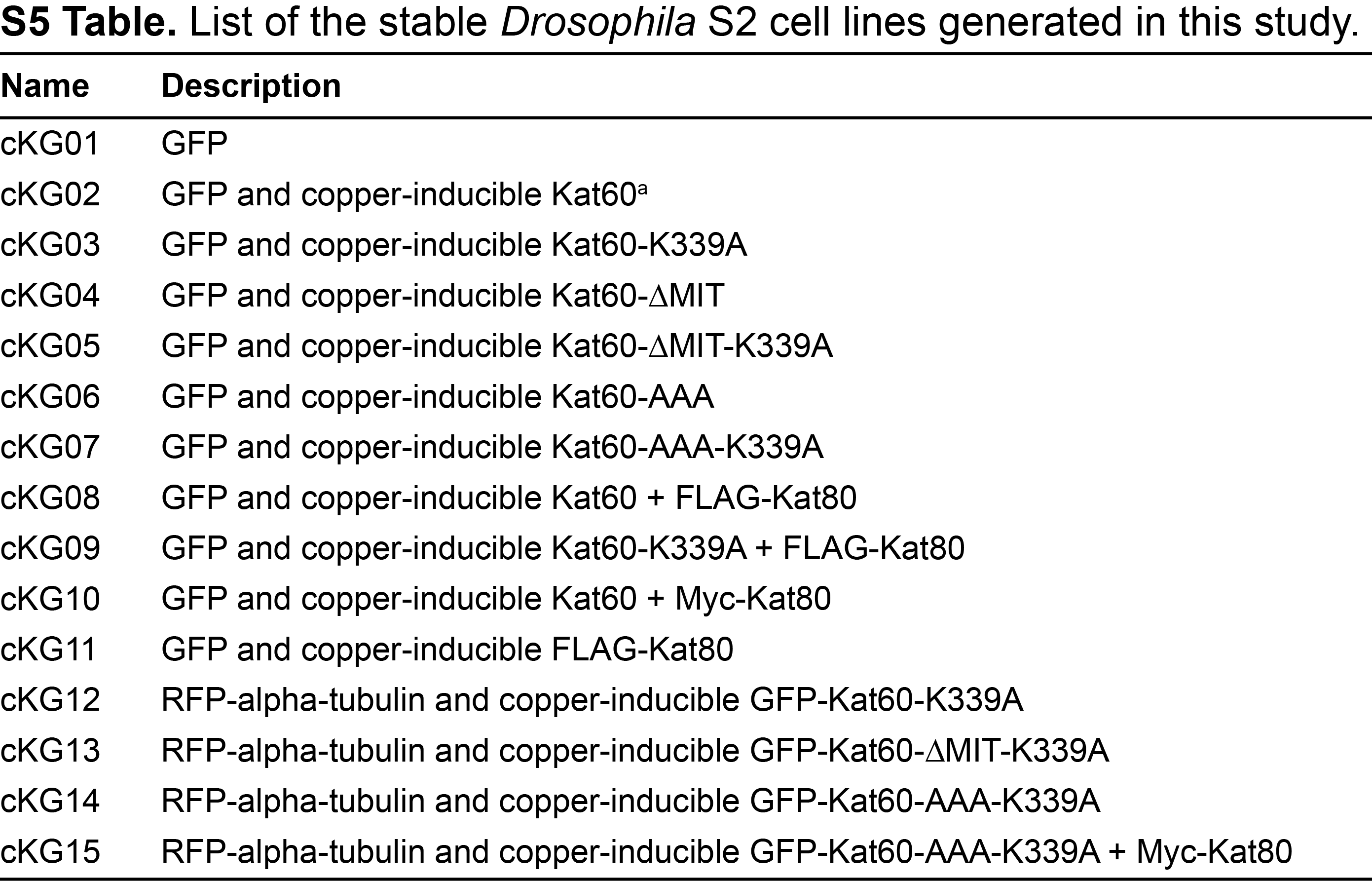

Supplement: S5 Table — aTwo independent Drosophila S2 cell lines stably expressing GFP and copper-inducible Kat60 were generated and used in this study. The first was used to acquire the data shown in S1 Fig and S3 Fig and the second was used to acquire the data shown in Fig 4, Fig 5, and Fig 6. (TIF) [file pone.0123912.s009.tif]

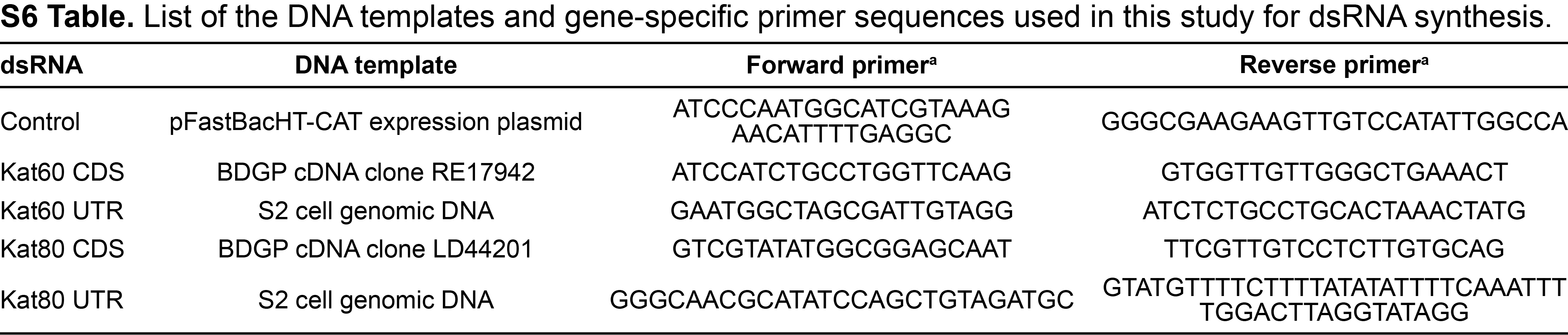

Supplement: S6 Table — aPrimer sequences are listed as 5’ to 3’ and each is preceded with the T7 promoter sequence 5’-TAATACGACTCACTATAGGG-3’. (TIF) [file pone.0123912.s010.tif]
